# Intestinal infections establish antigen-specific, long-lived memory CD4^+^ T cells in the brain and meninges

**DOI:** 10.1038/s41593-026-02428-4

**Published:** 2026-09-08

**Authors:** Aaron Fleming, Karen Neish, Rafael Di Marco-Barros, David A. Posner, Colin Y. C. Lee, Andrew Stewart, Zewen Kelvin Tuong, Miles Bremridge, Ana Peñalver, Mia Cabantous, Nathan Richoz, Anais Portet, Katherine Harcourt, Eleanor Gillman, Tammie Tao Min Sow, Tetsuo Hasegawa, David Ruano-Gallego, Gad Frankel, David Withers, Simon Clare, Menna R. Clatworthy

**Affiliations:** 1 Molecular Immunity Unit, University of Cambridge Department of Medicine, Cambridge, UK; 2 Cambridge Institute of Therapeutic Immunology and Infectious Diseases, University of Cambridge, Cambridge, UK; 3 Cellular Genetics, Wellcome Sanger Institute, Hinxton, UK; 4 Pathogen Genomics, Wellcome Sanger Institute, Hinxton, UK; 5 Department of Life Sciences, Imperial College London, London, UK; 6 Department of Molecular Biology, Centre of Molecular Biology Severo Ochoa, Madrid, Spain; 7 Centre for Immuno-Oncology, Nuffield Department of Medicine, University of Oxford, Oxford, UK

**Keywords:** Neuroimmunology, Central nervous system infections

## Abstract

The meninges form the border between the brain and periphery and house a rich network of immune cells. Here we show that gastrointestinal challenges (intracellular or extracellular bacteria and parasites) reshape the nature of CD4^+^ T cells in the dura mater, the outer meningeal layer, changing the dominant polarization states to T helper (T_H_) 1, T_H_17 and T_H_2 cells, respectively, with differing cytokine profiles. This occurs via CXCR6–CXCL16-dependent migration of gut-activated CD4^+^ T cells to the central nervous system, where they establish long-lived memory populations around the dural venous sinuses, within dural lymphoid aggregates and in the brain. Functionally, these orally primed dural CD4^+^ T were capable of rapid, antigen-specific recall responses, proliferating and producing cytokines upon intravenous rechallenge. Our findings reveal a direct link between intestinal and dural immunity, enabling the central nervous system borders to acquire immunological memory of gut microorganisms, a major source of bloodborne pathogens capable of reaching the brain via fenestrated dural vasculature.

## Abstract (web summary)

Intestinal CD4^+^ T cells migrate to the dura and brain following gut infection with bacteria or parasites, establishing a long-lived memory population capable of proliferation and cytokine production following rechallenge with bloodborne pathogens.

## Main

The meninges cover the brain and spinal cord and include three layers, the outer dura mater, the middle arachnoid mater and inner pia mater. The dura mater houses venous sinuses that drain blood from the underlying brain and overlying skull bone marrow and scalp, and this vascular network includes fenestrated endothelium, presenting a route by which circulating microorganisms may access the central nervous system (CNS)^1–3^. Commensurate with this threat, multiple populations of immune cells congregate around the dural venous sinuses^4–6^ and within dural-associated lymphoid tissue (DALT) aggregates^7^, including gut-derived IgA plasma cells^8^, and specific macrophage populations lie in close apposition to the meningeal vasculature^9^. Importantly, in addition to potential roles in defending the CNS from infectious challenges, meningeal immune cells may also have the capacity to influence neurons via cytokine production^10–13^.

Lymphocytes within DALT can sense and become activated by, circulating immune stimuli and intranasal neurotropic pathogens^7^. Whether immune stimuli in distant organs such as the gut alter dural immune cells is less clear. However, intestinal inflammation may be associated with CNS pathologies. For example, patients with inflammatory bowel disease (IBD) have an increased risk of depression and anxiety^14–17^. Intestinal inflammation, secondary to IBD or enteric infections, is associated with changes in CD4^+^ T cell polarization toward differing T helper (T_H_) cell states, a process influenced by antigen presenting cell-derived cytokines^18^, microbial metabolites^19,20^ and strength of TCR signal^21^. Depending on the context, this generates TBET^+^ IFN-γ-producing T_H_1 cells, GATA-3^+^ IL-4/IL-13-producing T_H_2 cells or RORγt^+^ IL-17/IL-22-producing T_H_17 cells, tailored to the prevalent immunological challenge^22^. Whether dural CD4^+^ T cell polarization may be influenced by gastrointestinal inflammation and infection is an important question, given the proven effects of T cell-derived cytokines on brain function. For example, in murine models, CD4^+^ T cell-derived IFN-γ can influence social behavior^10^, T cell-specific IL-4-deficiency results in impaired memory and cognition^11^ and γδ T cell-derived IL-17 improves short term memory^12^ but in excess can increase anxiety-like behavior^13^.

Here, we challenged mice with dextran sodium sulfate (DSS)-induced colitis, a model of IBD, oral extracellular and intracellular bacteria and schistosomiasis, a gut parasite, and assessed gut and dural CD4^+^ T cells. We found that dural CD4^+^ T cell polarization mirrored that of the intestine, with DSS colitis and *Citrobacter rodentium* challenge leading to an increase in dural IL-17-producing T_H_17 cells, whereas *Salmonella* challenge and schistosomiasis led to the appearance of dural T_H_1 and T_H_2 cells respectively. This change in dural CD4^+^ T cell population was dependent on Cxcr6–Cxcl16-mediated migration of cells from the gut to the CNS, with gut-activated, antigen-specific cells present in peri-sinus regions of the dura and in the extravascular space of the brain parenchyma. Gut-derived dural CD4^+^ T cells were capable of expansion and cytokine production upon rechallenge with the same antigen intravenously (i.v.). Our work reveals an important mechanism for gut–CNS communication that may contribute to CNS defense against circulating pathogens.

## Results

### Intestinal challenges shape the polarization and cytokine profile of dural CD4^+^ T cells

**Fig. 1:**
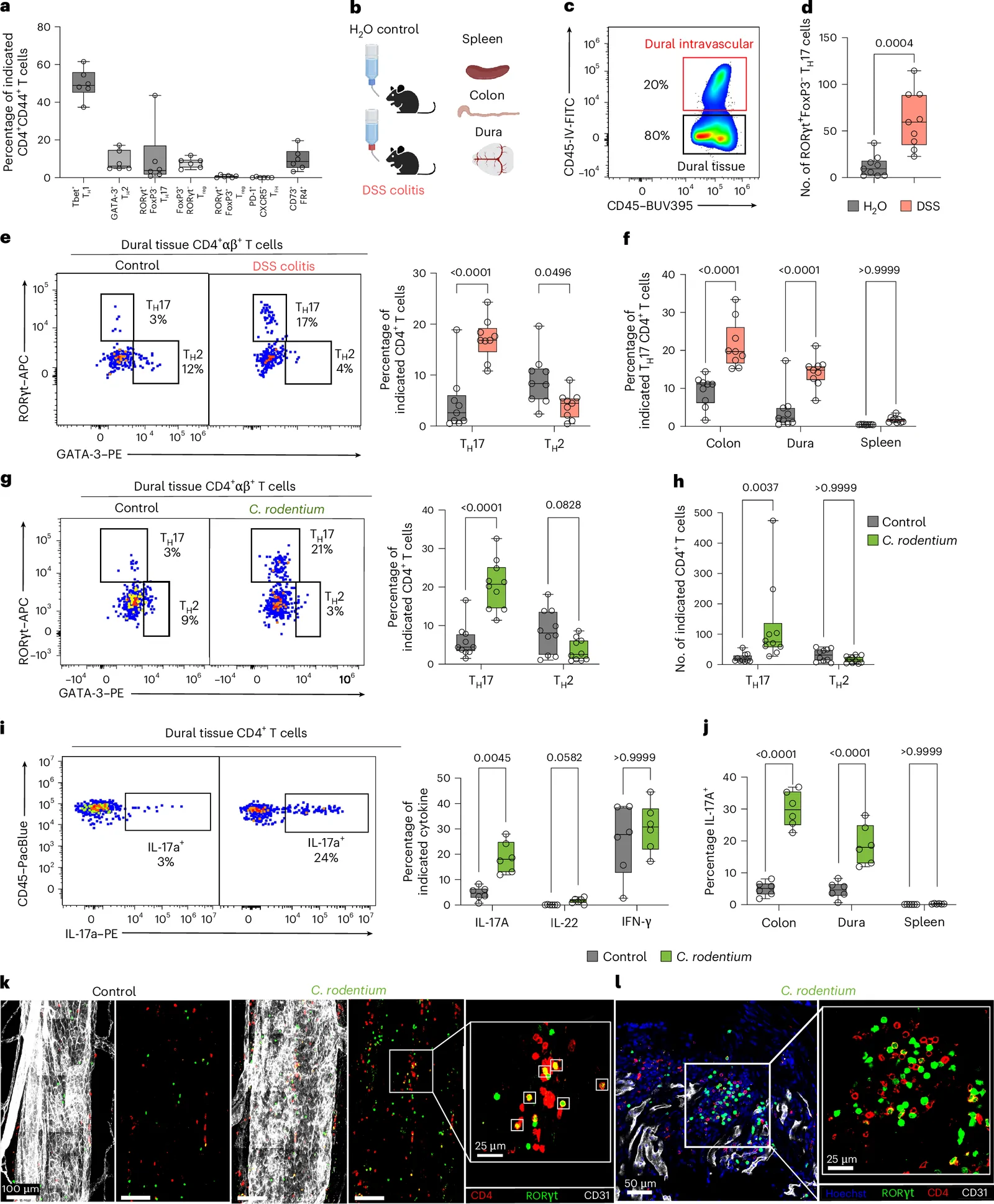
Dural T cells can be polarized by intestinal challenges to a T_H_17 phenotype. **a**, Cumulative box plots showing the distribution of CD4^+^ T_H_ cell subsets in the extravascular (CD45IV^neg^, not labelled with a CD45 antibody administered premortem) dural tissue of mice (*n* = 6). **b**, A schematic of the model to induce DSS colitis inflammation in mice. Mice receive control water or 2.5% DSS in drinking water for two periods of 5 days separated by a 14-day interval. Mice are killed 31 days after first DSS exposure with an anti-CD45 i.v. labelling antibody injected in the tail vein 5 min before killing and dura, colon and spleen collected. **c**, A representative flow cytometry plot used to discriminate between dural intravascular (CD45-IV^+^) or dural tissue (CD45-IV^−^) immune cells. **d**, The quantification of the total number of CD4^+^αβ^+^RORγt^+^ T_H_17 T cells in the tissue dural compartment of H_2_O control and DSS colitis mice (*n* = 9 per group). *P* values calculated using two-tailed Student’s unpaired *t*-test. **e**, Left: representative flow cytometry plots of T_H_ cell subsets (T_H_17, RORyt^+^GATA-3^−^; T_H_2, RORyt^−^GATA-3^+^) of total CD4^+^αβ^+^ T cells between H_2_O control and DSS colitis mice (*n* = 9 per group). Right: cumulative data of flow (*n* = 9 per group). *P* values calculated using two-way ANOVA with Bonferroni’s post test. **f**, The quantification of the percentage of tissue CD4^+^αβ^+^RORγt^+^ T_H_17 cells in the indicated organs between control and DSS colitis mice (*n* = 4 per group). *P* values calculated using two-way ANOVA with Bonferroni’s post test. **g**, The representative flow cytometry plots (left) or the cumulative box plots (right) showing percentage of T_H_ subsets of total CD4^+^αβ^+^ T cells in the dural tissue of control or *C. rodentium*-infected mice (*n* = 10 per group) 21 days post infection. *P* values calculated using two-way ANOVA with Bonferroni’s post test. **h**, The quantification of total number of CD4^+^αβ^+^RORγt^+^ T_H_17 T cells and αβ^+^CD4^+^GATA-3^+^ T_H_2 cells in the dural tissue compartment of control and *C. rodentium*-infected (*n* = 10 per group) mice 21 days post infection. *P* values calculated using two-way ANOVA with Bonferroni’s post-test. **i**, The representative flow cytometry plots (left) and the quantification (right) of percentage of indicated cytokine-expressing (IL-17a, IL-22 and IFN-γ) CD4^+^ T cells as a percentage of total CD4^+^ T cells in the dural tissue of control and *C. rodentium*-infected mice (*n* = 6 per group). *P* values calculated using multiple two-tailed Student’s unpaired *t*-tests and Bonferroni–Dunn multiple comparisons correction. **j**, The quantification (percentage) of IL-17a^+^ cells of total CD4^+^ T cells in the indicated organs between control and *C. rodentium*-infected mice (*n* = 6 per group). *P* values calculated using two-way ANOVA with Bonferroni’s post test. **k**, The whole mount confocal microscopy of the superior saggital sinus of duras from control (left) and *C. rodentium*-infected (right) RORγt–eGFP mice. T_H_17 cells are highlighted in white squares on far-right inset zoom. Representative images taken from a single mouse per group. **l**, The whole mount confocal microscopy of the rostral–rhinal venolymphatic hub of duras from *C. rodentium* infected RORγt–eGFP mice. Representative images taken from a single mouse. In **a** and **d**–**j**, the box plot’s center line shows the median, the box shows the IQR and the whiskers spans from the maximum to minimum values. Illustrations in **b** created in BioRender; Neish, K. https://biorender.com/442dkip (2026). Source data

**Fig. 2:**
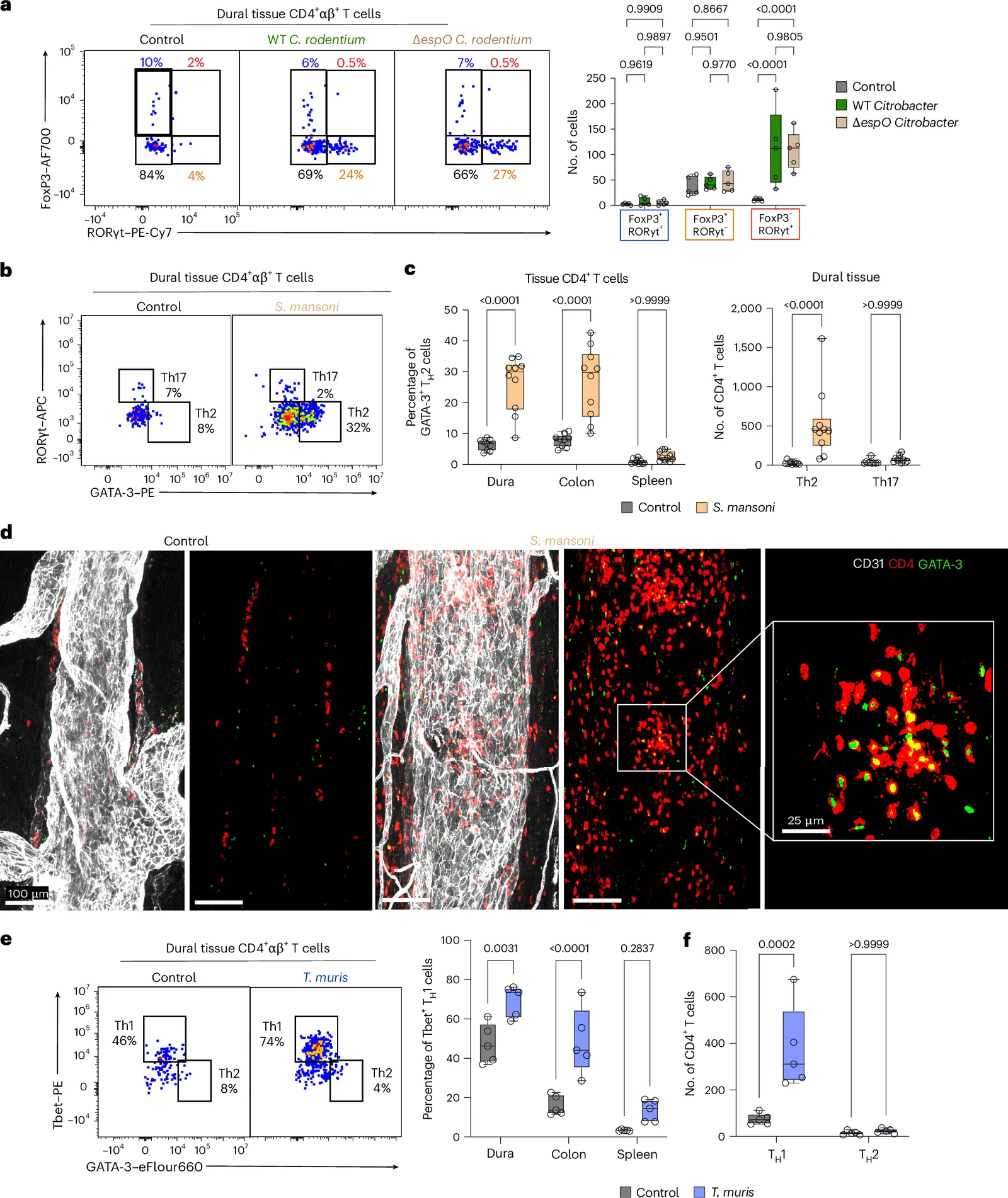
Dural T cells can be polarized by intestinal challenge to a T_H_2 or T_H_1 phenotype. **a**, Representative flow cytometry plots (left) and the quantification (right) of total number of CD4^+^αβ^+^ T_H_ cell subsets in control, WT *C. rodentium*-infected and Δ*espO* mutant *C. rodentium*-infected mice (*n* = 5 per group). *P* values calculated using two-way ANOVA with Bonferroni’s post test. **b**, Representative flow cytometry plots (left) and the quantification (right) of the percentage of T_H_ cell subsets (T_H_17, RORyt^+^GATA-3^−^; T_H_2, RORyt^−^GATA-3^+^) of total CD4^+^αβ^+^ T cells in the dural tissue-resident space between control mice and *S. mansoni*-infected mice (*n* = 9-10 per group). *P* values calculated using two-way ANOVA with Bonferroni’s post test. **c**, The quantification of the total number of T_H_2 or T_H_17 T cells in the tissue-resident dural space of control and *S. mansoni*-infected mice (*n* = 9 control group; *n* = 10 *S. mansoni* group). *P* values calculated using two-way ANOVA with Bonferroni’s post test. **d**, The whole mount confocal microscopy of the superior saggital sinus of duras from control (left) and *S. mansoni*-infected (right) mice. **e**, Representative flow cytometry plots (left) and the quantification (right) of the percentage of T_H_ cell subsets (T_H_1, T-bet^+^GATA-3^−^; T_H_2, T-bet^−^GATA-3^+^) of total CD4^+^αβ^+^ T cells in the dural tissue-resident space between control mice and *T. muris*-infected mice (*n* = 5 per group). *P* values calculated using two-way ANOVA with Bonferroni’s post test. **f**, The quantification of total number of T_H_1 or T_H_2 T cells in the tissue-resident dural space of control and *T. muris*-infected mice (*n* = 5 per group). *P* values calculated using two-way ANOVA with Bonferroni’s post test. In **a**, **c**, **e** and **f**, the box plot’s center line shows the median, the box shows the IQR and the whiskers spans from the maximum to minimum values. Source data

**Extended Data Fig. 1:**
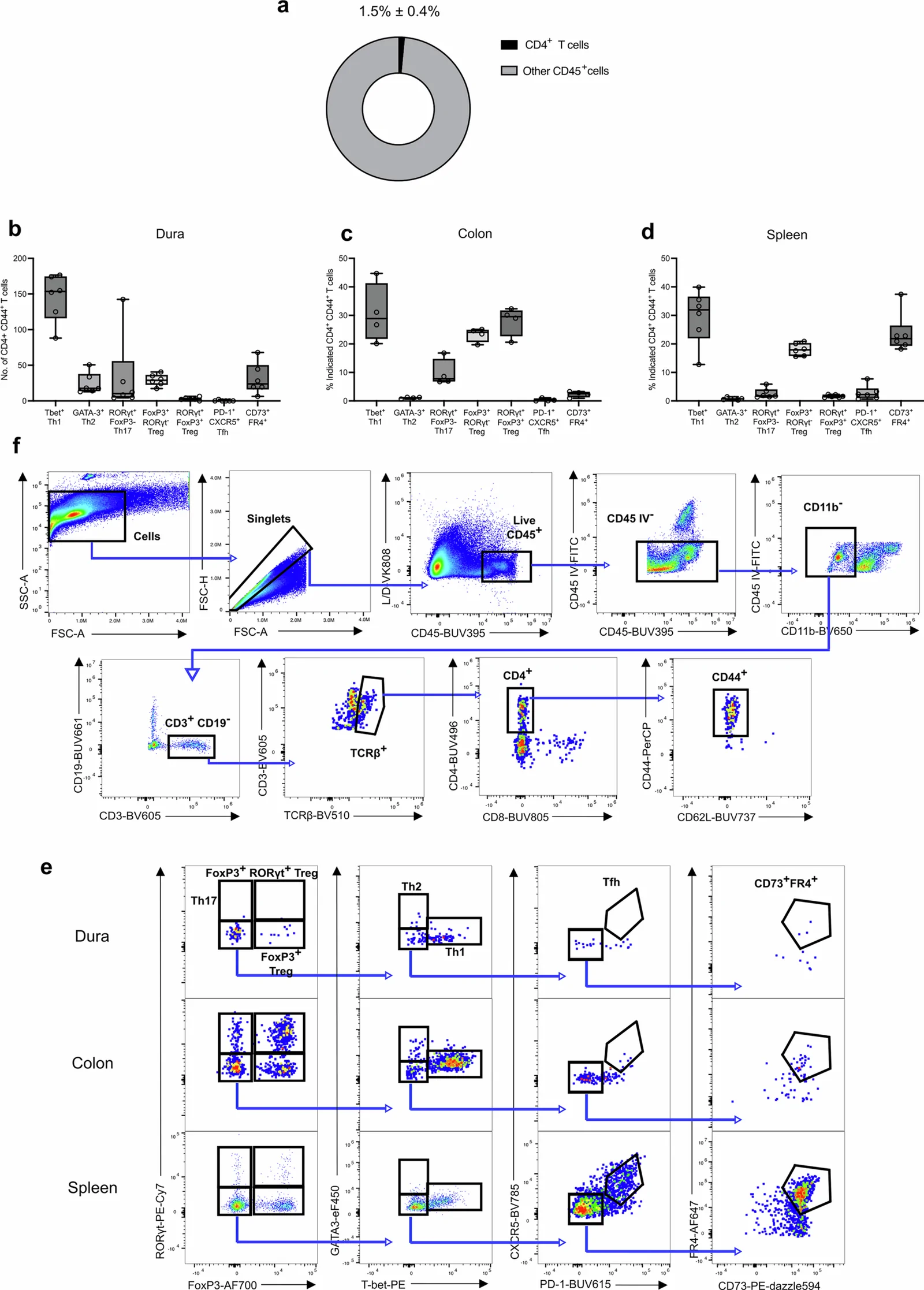
Dural CD4 T cell phenotyping and gating strategies. **A**: Pie chart showing percentage representation of CD4^+^ T cells in mouse dural tissue (CD45IV^neg^) amongst total CD45 live immune cells. Error bars represent SD. **B**: No. of indicated T helper cell subsets in dural tissue of mice (*n* = 5 per group). **C, D**: (c) Percentage of indicated T helper cell subsets in spleen tissue (d) or colon tissue (*n* = 5 per group). **E**: Representative flow gating strategy to identify dural tissue (CD45 IV^neg^) CD4^+^ T cells. **F**: Representative flow cytometry plots of tissue CD4^+^ T helper cell subsets in the indicated organs as defined by canonical transcription factor expression. **B-D**: The box plots centre line shows the median, the box shows the IQR, and the whiskers spans from the maximum to minimum values. Source data

**Extended Data Fig. 2:**
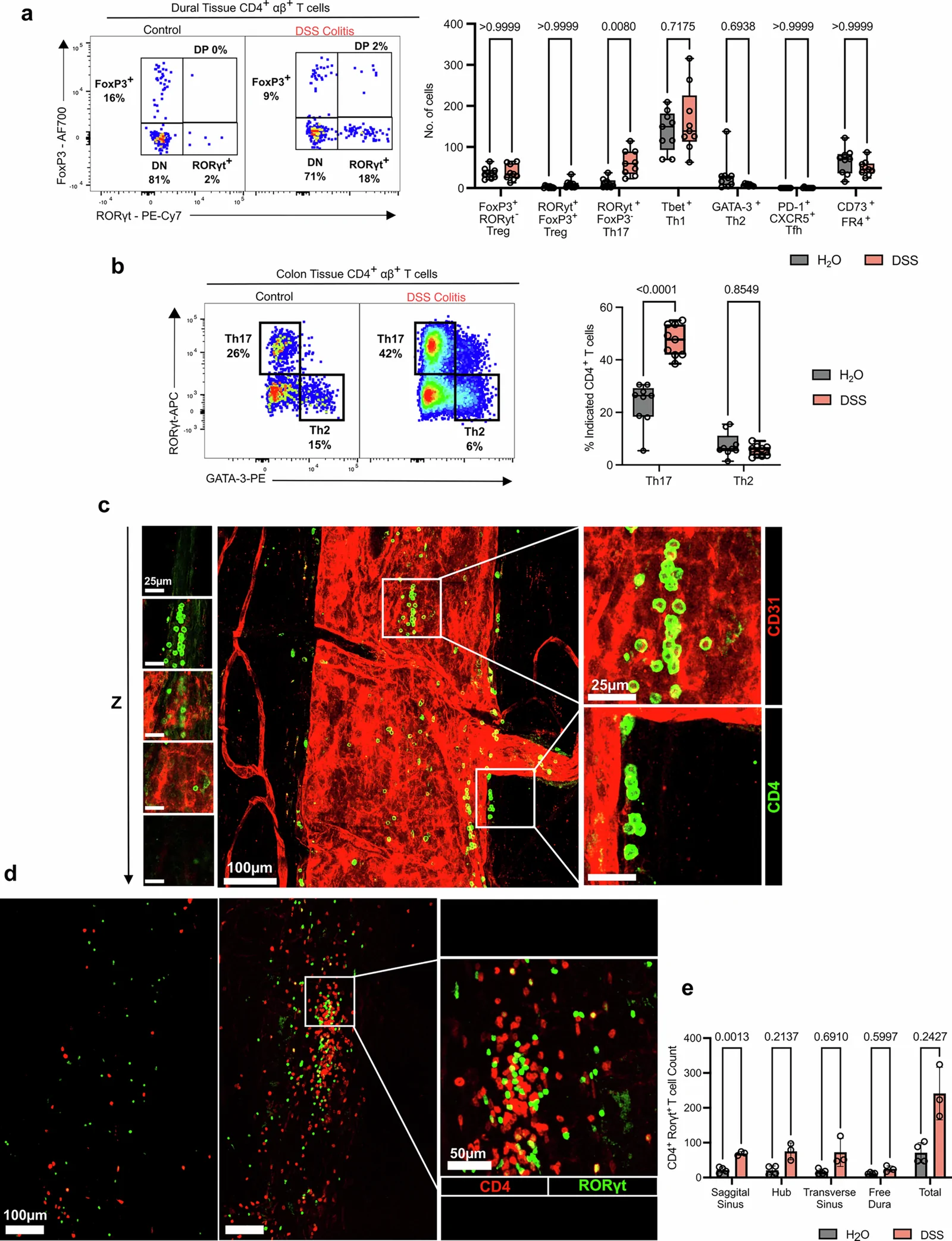
Dural Th17 cells are localised around dural venous sinuses upon intestinal challenge with DSS. **A:** Representative flow cytometry plots (left) and cumulative box plots (right) of dural tissue CD4^+^αβ^+^ T helper cell subsets in control and DSS colitis (*n* = 9 per group). *P* values calculated using multiple two-tailed Student’s unpaired t-tests and Bonferroni-Dunn multiple comparisons correction. **B:** Representative flow cytometry plots (left) and cumulative box plots (right) of colon tissue CD4^+^αβ^+^ Th17 or Th2 cells in control and DSS colitis (*n* = 4 per group). *P* values calculated using two-way ANOVA with Bonferroni’s post-test. **C**: Wholemount confocal imaging of dura showing localisation of CD4 T cells around the superior saggital sinus across the z-stack. **D, E:** (d) Whole mount confocal microscopy of the superior saggital sinus of dura from control (left) and DSS colitis (right). Th17 cells are highlighted in white squares on far-right inset zoom. (e) Manual quantification of CD4^+^ RORγt^+^ for indicated regions of the dura (control group *n* = 4; DSS group *n* = 3). Error bars represent SD. *P* values calculated using multiple two-tailed Student’s unpaired t-tests and Bonferroni-Dunn multiple comparisons correction. **A-B**: The box plots centre line shows the median, the box shows the IQR, and the whiskers spans from the maximum to minimum values. Source data

**Extended Data Fig. 3:**
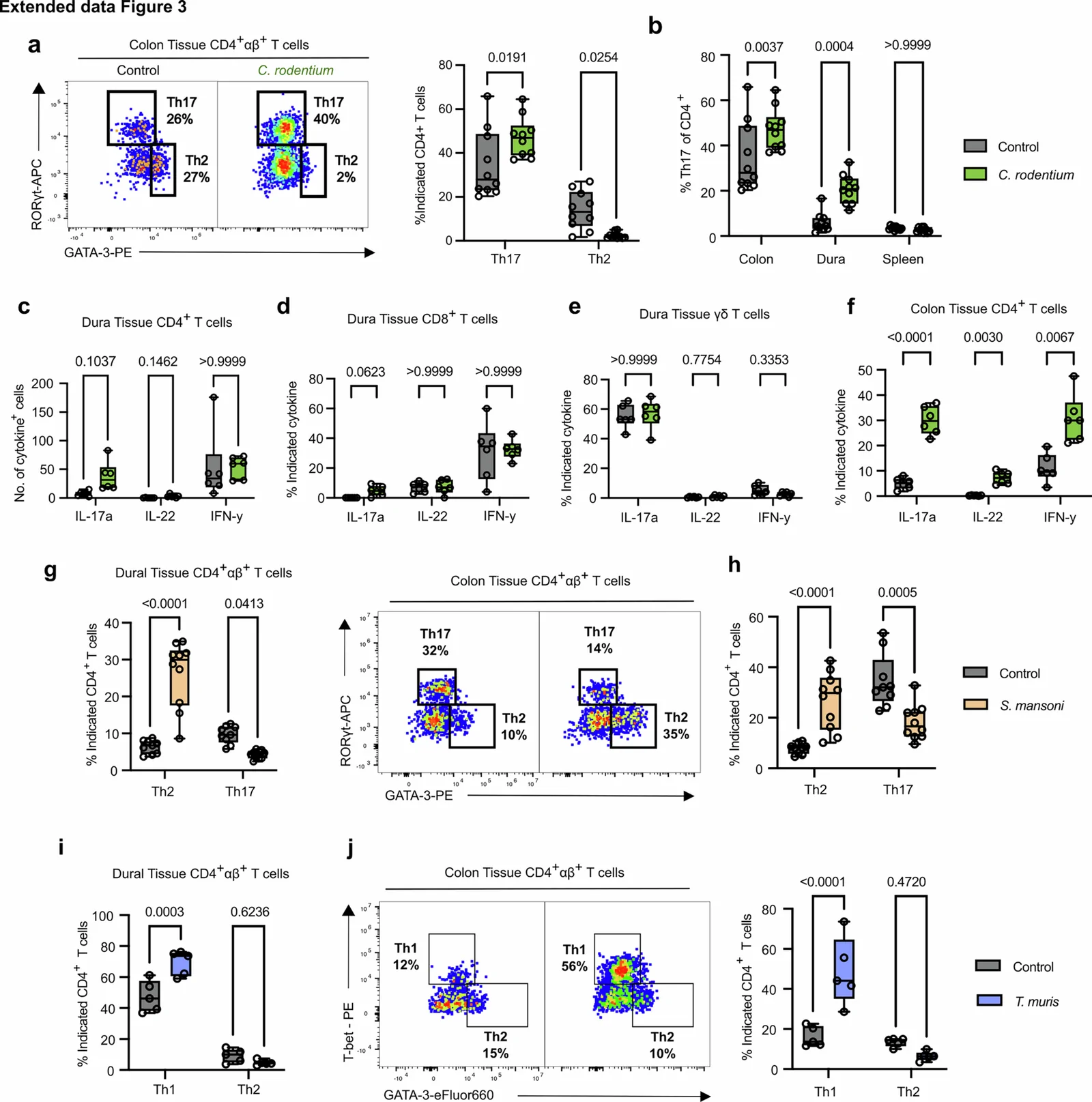
Dural T cells polarised to a Th2 or Th1 phenotype, depending on type of intestinal challenge. **A**: Representative flow cytometry plots (left) and cumulative box plots (right) of colon tissue CD4^+^αβ^+^ Th17 or Th2 cells in control and *C. rodentium*-infected mice 21 days post infection (*n* = 10 per group). *P* values calculated using two-way ANOVA with Bonferroni’s post-test. **B**: Quantification of the percentage of tissue CD4^+^ αβ^+^RORγt^+^ Th17 cells in the indicated organs between control and *C. rodentium*-infected mice 21 days post infection (*n* = 4 per group). *P* values calculated using two-way ANOVA with Bonferroni’s post-test. **C**: Quantification of indicated cytokine-expressing (IL-17a, IL-22, IFN-γ) CD4^+^ T cells in the dural tissue of control (*n* = 6) and *C. rodentium*-infected (*n* = 6) mice. *P* values calculated using multiple two-tailed Student’s unpaired t-tests and Bonferroni-Dunn multiple comparisons correction. **D-F**: Quantification of percentage of indicated cytokine-expressing (IL-17a, IL-22, IFN-γ) CD8^+^ T cells as a percentage of total CD8^+^ T cells (**D**) γδ^+^ T cells (**E**), and CD4^+^ T cells in the colon (**F**) in the dural tissue of control (*n* = 6) and *C. rodentium*-infected (*n* = 6) mice. *P* values calculated using multiple two-tailed Student’s unpaired t-tests and Bonferroni-Dunn multiple comparisons correction. **G:** Cumulative box plots of dural tissue CD4^+^αβ^+^ Th2 or Th17 cells in control (*n* = 9) and *S. mansoni*-infected (*n* = 10) mice 42 days post infection. *P* values calculated using two-way ANOVA with Bonferroni’s post-test. **H**: Representative flow cytometry (left) and cumulative box plots (right) of colon tissue CD4^+^αβ^+^ Th2 or Th17 cells in control (*n* = 9) and *S. mansoni*-infected (*n* = 10) mice 42 days post infection. *P* values calculated using two-way ANOVA with Bonferroni’s post-test. **I**: Cumulative box plots of dural tissue CD4^+^αβ^+^ Th1 or Th2 cells in control and *T. muris*-infected mice 21 days post infection (*n* = 5 per group). *P* values calculated using two-way ANOVA with Bonferroni’s post-test. **J**: Representative flow cytometry (left) and cumulative box plots (right) of colon tissue CD4^+^αβ^+^ Th1 or Th2 cells in control and *T. muris-*infected mice 21 days post infection (*n* = 5 per group). *P* values calculated using two-way ANOVA with Bonferroni’s post-test. **A-J**: The box plots centre line shows the median, the box shows the IQR, and the whiskers spans from the maximum to minimum values. Source data

Given that the key cellular effectors of dural humoral memory, antibody-secreting plasma cells, may be educated in the gut^8^, we hypothesized that gut-activated and polarized CD4^+^ T cells, the orchestrators of cellular adaptive immunity, may similarly relocate to the dura. In steady state, αβ CD4^+^ T cells formed a minority population among dural immune cells (Extended Data Fig. 1a) and were predominantly non-naive, with around half of cells expressing Tbet (Fig. 1a and Extended Data Fig. 1b–f). Following oral challenge with DSS (Fig. 1b), an osmotic colitogen that induces epithelial barrier breach and expansion of intestinal T_H_17 cells^23^, we observed a significant increase in RORγt^+^ T_H_17 cells in the colon (as expected), with no change in Foxp3^+^ T cells, T_H_1, T_H_2, T follicular helper (T_FH_) or CD73^+^FR4^+^CD4^+^ T cells (Fig. 1c–f and Extended Data Fig. 2a). This expansion in colonic T_H_17 cells was mirrored by an increase in T_H_17 cells within the extravascular compartment of dura, in aggregates around the venous sinuses and in rostral–rhinal DALT, with no significant change in T_H_17 cells in the intravascular dural compartment or spleen (Fig. 1c–f and Extended Data Fig. 2a–e). Similarly, 21 days after oral challenge with *C. rodentium*, an attaching–effacing pathogen known to induce intestinal T_H_17 cells^24^, we observed a significant increase in colonic and extravascular dural RORγt-expressing CD4^+^ T cells (Fig. 1g,h and Extended Data Fig. 3a,b). Dural T_H_17 cells were predominantly IL-17-producing, with little intracellular IL-22 identified (Fig. 1i,j and Extended Data Fig. 3c). This increase in IL-17a expressing cells was specific to CD4^+^ T cells and was not observed in the γδ or CD8^+^ T cell compartments within the dura (Extended Data Fig. 3d–f). Spatially, T_H_17 cells were located along the dural venous sinus and within rostral–rhinal DALT (Fig. 1k,l). Of note, in mice challenged orally with an attenuated mutant *C. rodentium* strain (Δ*EspO*) that induces less intestinal IL-22 secretion and neutrophil recruitment^25^, we observed a similar level of expansion of dural T_H_17 cells compared with mice challenged with wild-type (WT) *C. rodentium* (Fig. 2a), together suggesting that CD4^+^ T cells activated in the intestine relocate to the dura.

To determine if the linked expansion of gut and dural CD4^+^ T cells was limited to the context of a T_H_17-polarizing intestinal stimulus, we next challenged mice with the helminth *Schistosoma mansoni*^26^. *S. mansoni* is a worm with a complex life cycle, initially infecting via cercariae, which burrow into the skin, transform into schistosomula and then enter the vasculature and migrate to the portal system, where they mature into adult worms. Eggs released by female parasites within the vasculature extravasate traverse the intestinal wall and ultimately cross the epithelium into the lumen of the intestine where they induce a strong T_H_2 response^27^. At 42 days following challenge with *S. mansoni*, we found a marked expansion of Gata-3-expressing T_H_2 cells in the intestine, with a similar expansion observed in the dura, but little change in splenic T_H_2 cells (Fig. 2b,c and Extended Data Fig. 3g,h). Confocal imaging of dural wholemounts confirmed a substantial increase in CD4^+^ T cells, particularly GATA-3-expressing cells, in aggregates along the walls of the venous sinuses (Fig. 2d). By contrast, following oral challenge and the establishment of gut-limited chronic infection with *Trichuris muris* (mouse whipworm, known to induce a dominant T_H_1 response in C57BL/6 mice^28^), an increase in Tbet-expressing T_H_1 cells number was present in the colon and dura (Fig. 2e,f and Extended Data Fig. 3i,j).

Altogether, our data show that the nature of an orally administered intestinal infection not only determines gut CD4^+^ T cell polarization but also can profoundly alter the polarization of tissue, extravascular dural αβ CD4^+^ T cells.

### Intestinal challenge leads to accumulation of antigen-specific CD4^+^ T cells in the dura

**Fig. 3:**
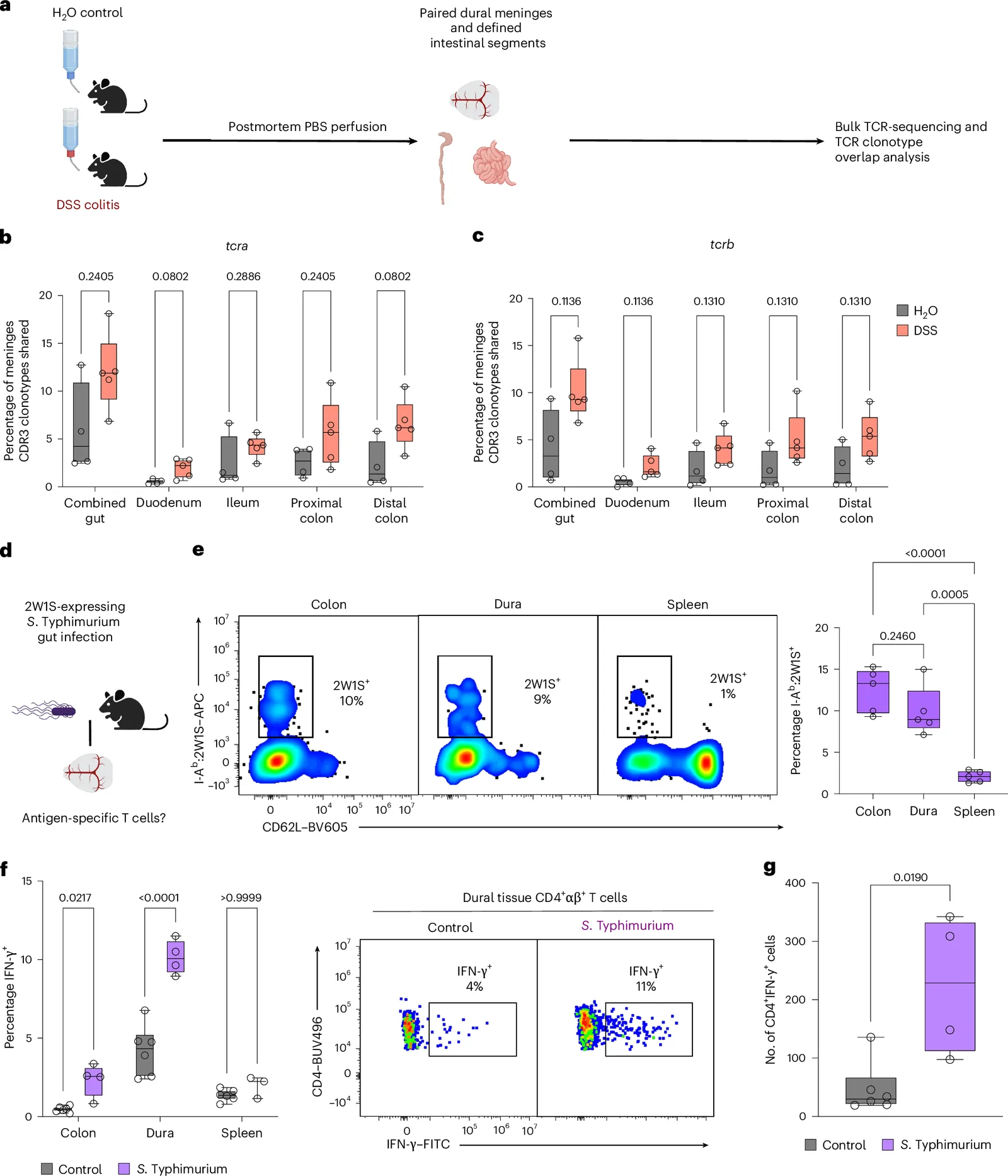
Intestinal challenge leads to expansion of antigen-specific CD4^+^ T cells in the dura. **a**, A schematic of experimental setup for the TCR clonotype analysis in DSS colitis. Mice were given either control water or 2.5% DSS in their drinking water for 7 days and killed 14 days later with PBS perfusion. Paired dural meninges or defined intestinal segments were collected for TCR-sequencing analysis. **b**,**c**, The quantification of percentage of meninges CDR3 clonotypes shared with the specified intestinal segments as measured by *tcra* (**b**) or *tcrb* (**c**) (*n* = 4 control group; *n* = 5 DSS group). *P* values calculated using multiple two-tailed Mann–Whitney *U* tests with false discovery rate multiple testing using the two-stage step-up method. **d**, The experimental setup to identify the presence of antigen-specific CD4^+^ T cells in response to oral infection with *S*. Typhimurium-2W1S. **e**, Representative flow cytometry plots (left) or the quantification (right) of percentage of 2W1S antigen-specific CD4^+^ T cells in the specified organs 21 days post infection with *S*. Typhimurium-2W1S (*n* = 5 per group). *P* values calculated using ordinary one-way ANOVA with Tukey’s multiple comparisons test. **f**, The quantification (left) and representative flow cytometry plots (right) of percentage of dural tissue CD4^+^ αβ^+^ T cells expressing IFN-y in control mice and mice infected orally with *S*. Typhimurium-2W1S 21 days post infection (*n* = 6 control group; *n* = 4 *S*. Typhimurium group). *P* values calculated using two-way ANOVA with Bonferroni’s post test. **g**, The quantification of total number of dural tissue CD4^+^αβ^+^ T cells expressing IFN-y in in control mice and mice infected orally with *S*. Typhimurium-2W1S 21 days post infection (*n* = 6 control group; *n* = 4 *S*. Typhimurium group). *P* values calculated using two-tailed Mann–Whitney *U* test. In **b**, **c** and **e**–**g**, the box plot’s center line shows the median, the box shows the IQR and the whiskers spans from the maximum to minimum values. Illustrations in **a** and **d** created in Biorender; Neish, K. https://biorender.com/7etshl6 (2026). Source data

**Extended Data Fig. 4:**
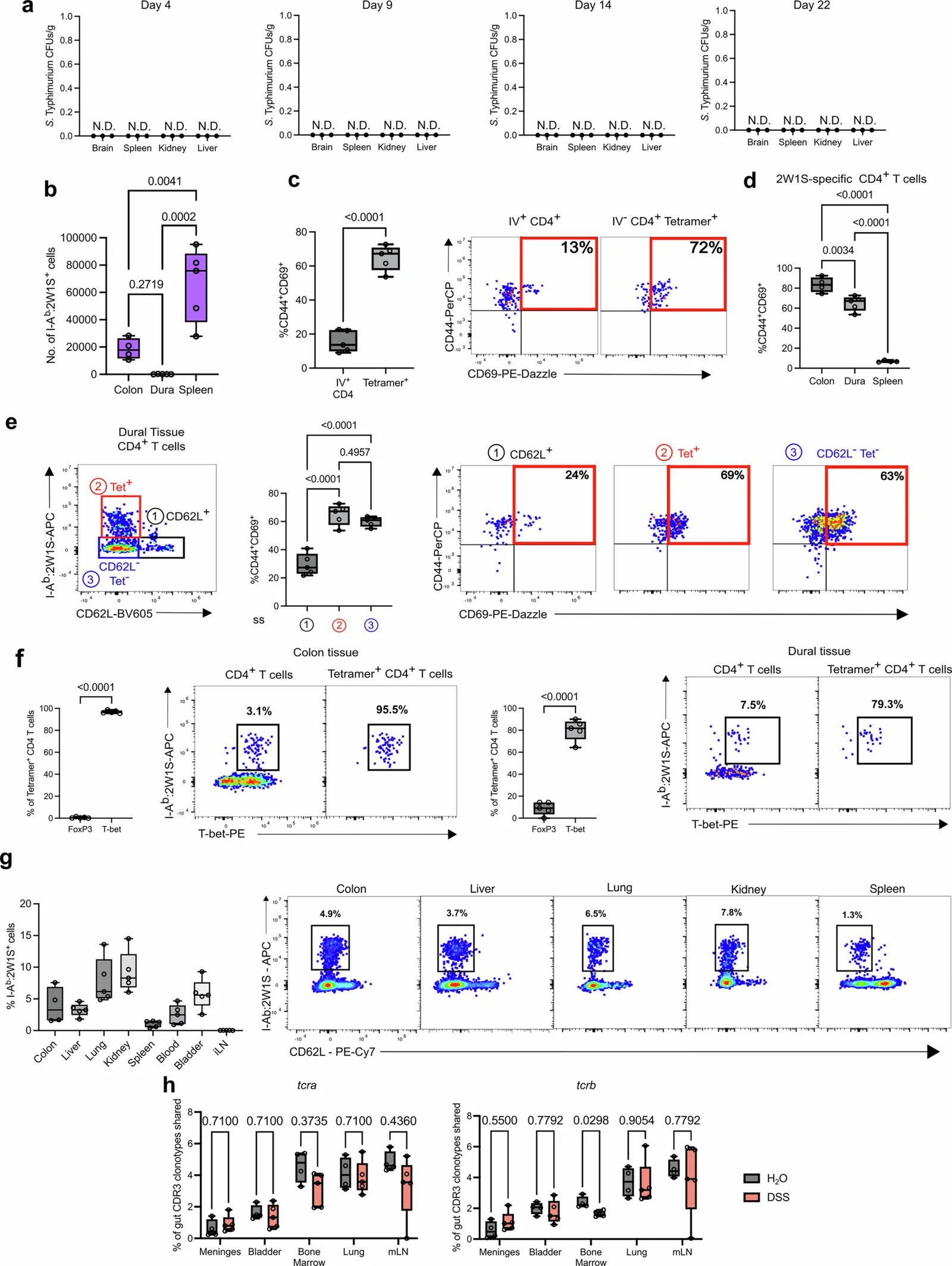
Intestinal challenge leads to CD44^+^ CD69^+^ antigen-specific CD4 T cells in the dura. **A:** *S*. Typhimurium CFUs in indicated peripheral organs at different timepoints following oral challenge (*n* = 3 per group, per day). **B:** Quantification of number of 2W1S-specific CD4^+^ T cells in the colon (*n* = 4), dura (*n* = 5), and spleen (*n* = 5) following oral *S*. Typhimurium infection. *P* values calculated using ordinary one-way ANOVA with Tukey’s multiple comparisons test. **C:** Quantification (left) and representative flow cytometry (right) of co-expression of CD44 and CD69 (CD44^+^CD69^+^) differences between dural intravascular CD4^+^ T cells (“IV^+^ CD4^+^) and dural tissue 2W1S-specific CD4^+^ T cells following oral *S*. Typhimurium infection (“IV^−^ CD4^+^ tetramer^+^).(*n* = 5 per group). *P* values calculated using Student’s unpaired two-tailed *t*-test. **D**: Quantification (top) and representative flow cytometry (bottom) of dural tissue CD4^+^ T cell co-expression of CD44 and CD69 (CD44^+^CD69^+^) in the specified subsets (1 = naive T cells CD62L^−^, 2 = 2W1S antigen-specific, 3 = non-2W1S non-naive T cells). (*n* = 5 per group). *P* values calculated using ordinary one-way ANOVA with Tukey’s multiple comparisons test. **E**: Quantification of 2W1S antigen-specific CD4^+^ T cells co-expression of CD44 and CD69 (CD44^+^CD69^+^) across the indicated organs. (*n* = 5 per group). *P* values calculated using ordinary one-way ANOVA with Tukey’s multiple comparisons test. **F:** Quantification (left) of T-bet or FoxP3 expression on 2W1S-specific CD4^+^ T cells in the colon and dura. Representative flow cytometry (right) comparing T-bet expression in overall CD4^+^ T cells and 2W1S-specific CD4^+^ T cells in the indicated tissues. (*n* = 5 per group). *P* values calculated using two-tailed Student’s unpaired *t*-test. **G:** Percentage (left) and representative flow cytometry (right) of 2W1S-specifc CD4^+^ T cells in peripheral organs 21 days following oral *S*. Typhimurium-2W1S infection). (*n* = 4 colon; *n* = 5 all other tissues). **H:** Quantification of percentage of gut CDR3 clonotypes shared with the specified tissues as measured by *tcra* (left) or *tcrb* (right). (n = 4 control group; *n* = 5 DSS group). *P* values calculated using multiple two-tailed Mann-Whitney U tests with false discovery rate multiple testing using the two-stage step-up method. **B-H**: The box plots centre line shows the median, the box shows the IQR, and the whiskers spans from the maximum to minimum values. Source data

To further probe the relationship of T cells in the gut and dura, we performed T cell receptor (TCR) sequencing on paired samples of dura, duodenum, ileum, proximal and distal colon 21 days following the induction of DSS colitis (Fig. 3a). We found a significant increase in the proportion of identical TCRs shared between dura and duodenum and distal colon in DSS mice compared with water-treated controls (Fig. 3b,c), suggesting that clones expanded in the intestine may seed to the dura. To test this further, we orally challenged mice with an attenuated 2w1s peptide-expressing *Salmonella enterica* serovar Typhimurium (*S*. Typhimurium), which we confirmed is intestinal-limited^29^ (Extended Data Fig. 4a). At 21 days following infection, 2w1s-specific, CD62L-negative CD4^+^ T cells were expanded in the extravascular compartment of both colon and dura and, proportionally, to a lesser extent in the spleen (Fig. 3d,e and Extended Data Fig. 4b). These dural CD4^+^ T cells were CD44^+^, expressed the tissue-residency marker CD69 (Extended Data Fig. 4c–e) and were IFN-γ-secreting (Fig. 3f,g and Extended Data Fig. 4f). Together, these data support the conclusion that intestinal-polarized, antigen-specific CD4^+^ T cells relocate to the dura to establish a tissue memory population at this CNS border tissue following gut challenge. We were also able to identify gut-derived 2w1s-specific CD4^+^ T cells in the extravascular compartment of liver, lung, kidney and bladder following intestinal challenge with attenuated 2w1s-*S*. Typhimurium (Extended Data Fig. 4g) and shared TCR clones between the gut and these organs (Extended Data Fig. 4h).

### Dural relocation of intestinal CD4^+^ T cells is dependent on Cxcr6–Cxcl16 interactions

**Fig. 4:**
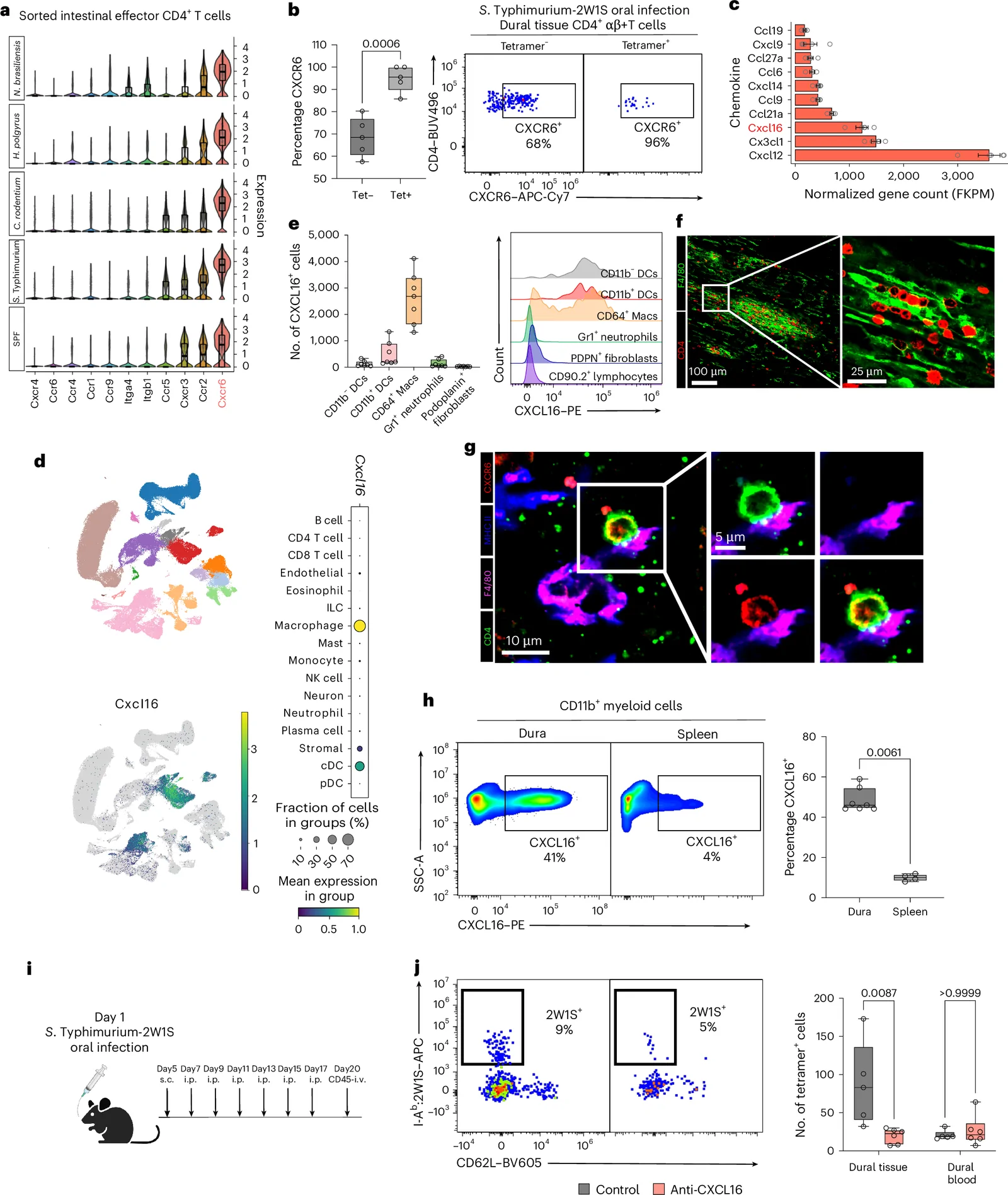
Dural relocation of intestinal CD4^+^ T cells is dependent on Cxcr6–Cxcl16 interactions. **a**, The box plots of expression in scRNA-seq data of the top ten most highly expressed chemokine receptors in effector (*Cd44*^+^*Sell*^−^*Ccr7*^−^*Foxp3*^−^) cells from sorted colonic CD4^+^ T cells from mice that underwent the indicated oral intestinal infections^30^. **b**, The quantification (left) or representative flow cytometry plots (right) of Cxcr6 expression on dural tissue 2W1S tetramer^+^ or tetramer^−^ CD4^+^ T cells in mice infected with 2W1S-expressing *S*. Typhimurium (*n* = 5 per group). *P* values calculated using two-tailed Student’s unpaired *t*-test. **c**, Bar plots of expression in bulk RNA-seq data of dural meninges of most highly expressed chemokines (*n* = 5). Error bars represent s.d. FKPM, fragments per kilobase of transcript per million fragments mapped. **d**, Top left: the UMAP of 130,908 cells from an integrated scRNA-seq atlas of meningeal tissue in mice (https://www.cnsbordercellatlas.org/), colored by cell type. Bottom left: the UMAP of *cxcl16* expression or (right) dot plots showing *cxcl16* expression in various cell types. ILCs, innate lymphoid cells; cDC, conventional dendritic cell; pDC, plasmacytoid dendritic cell. **e**, The quantification of total number of Cxcl16^+^ cells by cell types (left) or histograms (right) showing Cxcl16 expression (*n* = 7 per group). DCs, dendritic cells; Macs, macrophages. **f**,**g**, The whole mount confocal microscopy of the dura showing CD4^+^ T cells colocalized with F4/80^+^ macrophage (**f**) and Cxcr6^+^CD4^+^ T cells interacting with F4/80^+^ macrophages (**g**). Representative images taken from a single mouse. **h**, The representative flow cytometry plots (left) and cumulative data (right) showing percentage Cxcl16 expression in CD11b^+^ cells between dura and spleen (*n* = 7 dura; *n* = 4 spleen). *P* values calculated using Mann–Whitney *U* test. **i**, A schematic of experimental setup. Mice were orally infected with 2W1S-expressing *S*. Typhimurium and received either control isotype antibody or neutralizing anti-Cxcl16 antibody at the indicated time points in the indicated manner. **j**, The quantification (left) or representative flow cytometry plots (right) of the total number of dural tissue or dural blood CD4^+^ antigen-specific T cells between control or anti-Cxcl16-treated oral *S*. Typhimurium-infected mice (*n* = 5 control group; *n* = 6 anti-Cxcl16 group). *P* values calculated using multiple two-tailed Mann–Whitney *U* test with Bonferonni–Dunn multiple comparisons correction. In **b**, **e**, **h** and **j**, the box plot’s center line shows the median, the box shows the IQR and the whiskers spans from the maximum to minimum values. Illustrations in **i** created in BioRender; Neish, K. https://biorender.com/ts44rv3 (2026). Source data

**Extended Data Fig. 5:**
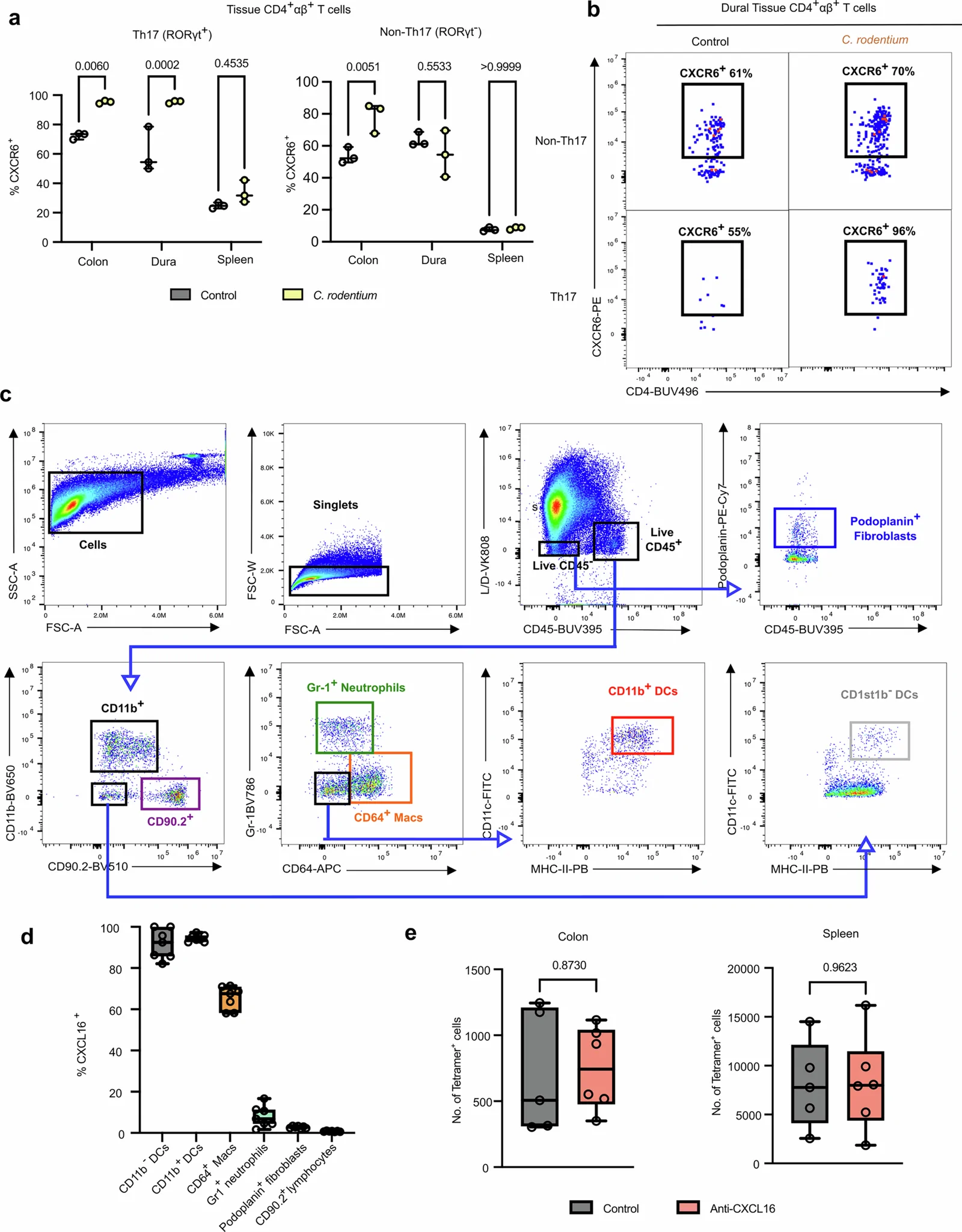
Meningeal APCs are a major source of CXCL16. **A:** Quantification of CXCR6 expression on Th17 (left) and non-Th1 (right) tissue CD4^+^ T cells in the specified organs in control mice and mice receiving an oral *C. rodentium* infection 21 days post infection (*n* = 3 per group). (*n* = 3 per group). *P* values calculated using two-way ANOVA with Bonferroni’s post-test. Error bars represent the minimum and maximum values per group. **B**: Representative flow cytometry plots of dural CD4^+^ non-Th17 (top) and Th17 (bottom) T cells in control mice and mice receiving an oral *C. rodentium* infection 21 days post infection. **C**: Representative gating strategy for identifying dural myeloid, lymphoid, and fibroblasts populations for CXCL16 staining. **D**: Quantification of percentage of specified cells expressing CXCL16 in dural tissue (*n* = 7 per group). **E**: Quantification of numbers of 2W1S-specific CD4 + T cells in the colon (left) and spleen (right) of mice orally infected with *S*. Typhimurium-2W1S receiving either control or anti-CXCL16 treatment (*n* = 5 control group; *n* = 6 anti-CXCL16 group). *P* values calculated using paired two-tailed t-test. **D-E**: The box plots centre line shows the median, the box the IQR, and the whiskers the minimum and maximum values. Source data

**Extended Data Fig. 6:**
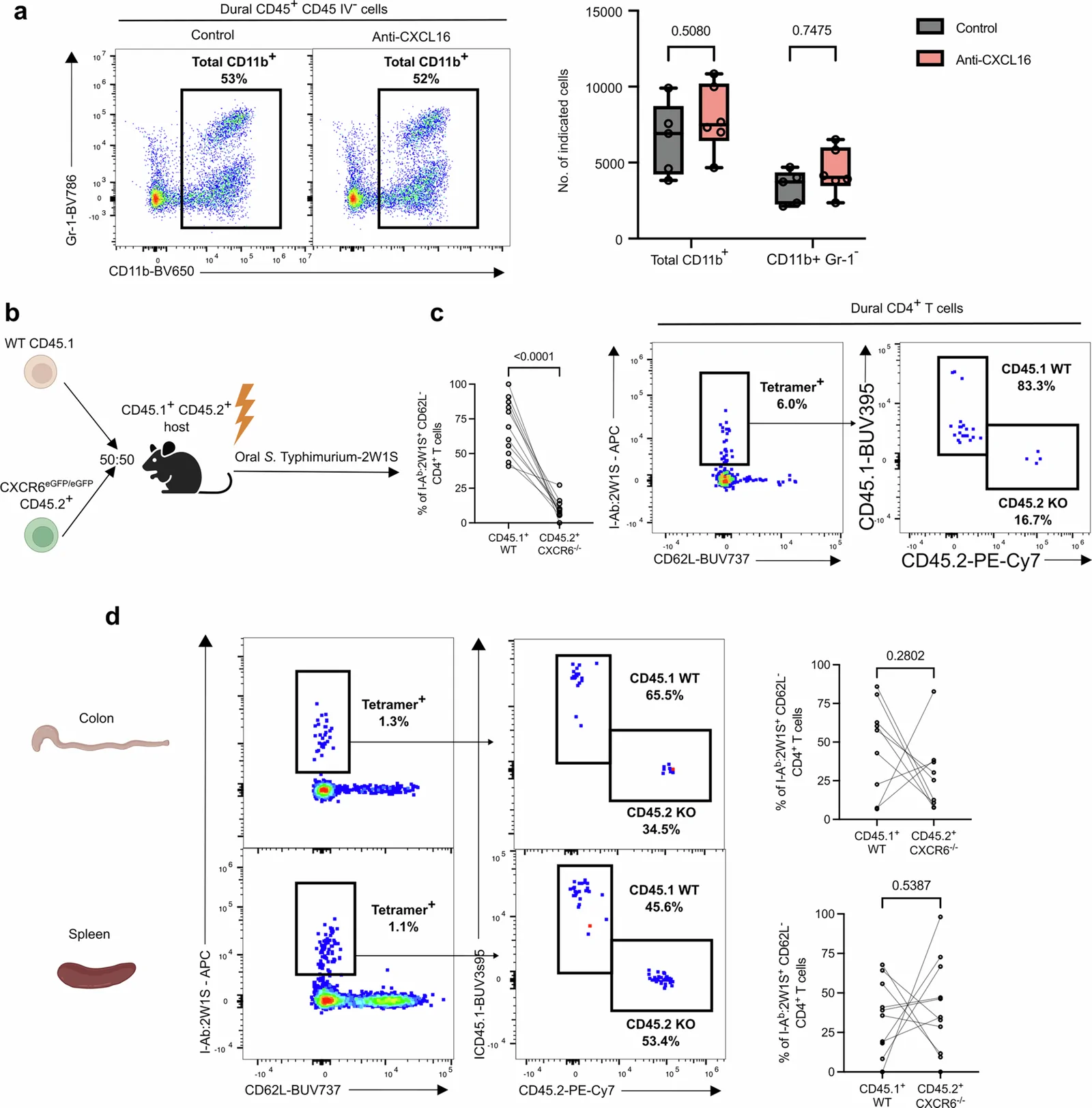
CXCR6-deficient antigen-specific CD4 T cells exhibited impaired recruitment to the dura. **A:** Representative flow cytometry (left) and quantification (right) of the number of total CD11b^+^ myeloid cells, and CD11b^+^ Gr-1^−^ myeloid cells between control or anti-CXCL16-treated oral *S*. Typhimurium-infected mice (*n* = 5 control group; *n* = 6 anti-CXCL16 group). The box plots centre line shows the median, the box shows the IQR, and the whiskers spans from the maximum to minimum values. *P* values calculated using two-way ANOVA with Bonferroni’s post-test. **B:** Schematic of experimental layout. CD45.1 × CD45.2 host mice were irradiated and reconstituted with 50:50 with CD45.1 WT: CD45.2 CXCR6^−/−^ bone marrow. After reconstitution, the mice were orally infected with *S*. Typhimurium-2W1S. **C:** Quantification (left) and representative flow cytometry (left) of 2W1S-specific CD4 + T cells between the two genotypes. (*n* = 11). *P* values calculated using paired two-tailed Wilcoxon test. **D:** Representative flow cytometry (left) and quantification (right) of 2W1S-specific CD4 + T cells between the two genotypes in colon and spleen. (*n* = 10). *P* values calculated using paired two-tailed t-test. Illustrations in **b** and **d** created in BioRender; Neish, K. https://biorender.com/br7dsuu (2026). Source data

To identify the chemokine–chemokine receptor signals involved in the localization of intestinal CD4^+^ T cells into the dura, we examined chemokine receptor expression in a published single-cell RNA sequencing (scRNA-seq) data generated from sorted intestinal CD4^+^ T cells^30^. *Cxcr6* was the most highly expressed chemokine receptor in gut CD4^+^ T cells, regardless of the nature of the T cell polarizing stimulus (Fig. 4a). We confirmed Cxcr6 surface expression on both intestinal and dural T_H_17 cells following *C. rodentium* infection (Extended Data Fig. 5a,b). Indeed, the tetramer-positive CD4^+^ T cells present in the dura following oral challenge with 2w1s-expressing *S*. typhimurium were almost universally Cxcr6^+^ (Fig. 4b). Within the dura, the Cxcr6 ligand, *Cxcl16*, was among the most highly expressed chemokine transcripts (Fig. 4c), identifying Cxcr6–Cxcl16 as a potential axis mediating the recruitment of gut-activated CD4^+^ T cells to the dura. Using our integrated scRNA-seq dataset of CNS border tissues^31^, we identified meningeal macrophages as the major cell type expressing *Cxcl16*, with more limited expression in conventional dendritic and stromal cells (Fig. 4d), confirmed at a protein level (Fig. 4e and Extended Data Fig. 5c,d). Indeed, Cxcr6^+^CD4^+^ T cells spatially colocalized with dural macrophages (Fig. 4f, g). In contrast to dura, there was limited expression of Cxcl16 in splenic macrophages (Fig. 4h). To test the importance of this axis in dural CD4^+^ T cell recruitment, we administered a Cxcl16-blocking antibody from days 5–17 following 2w1s-*S*. Typhimurium challenge (Fig. 4i). This led to a reduction in the number of antigen-specific CD4^+^ T cells recruited to the dura (Fig. 4j) but not the colon or spleen (Extended Data Fig. 5e), with no effect on the number of dural CD11b^+^ myeloid cells (Extended Data Fig. 6a). Similarly, using mixed bone marrow chimeras, we found significantly attenuated recruitment of *Cxcr6*-deficient tetramer-positive CD4^+^ T cells to the dura compared with WT CD4^+^ T cells following oral *S*. Typhimurium challenge (Extended Data Fig. 6b–d). Altogether, this confirms that the increase in dural CD4^+^ T cells observed following intestinal challenge occurs due to cellular immigration in a Cxcr6–Cxcl16 dependent manner.

### Intestinal challenge leads to long-lived dural antigen-specific memory CD4^+^ T cells capable of a recall response upon i.v. rechallenge

**Fig. 5:**
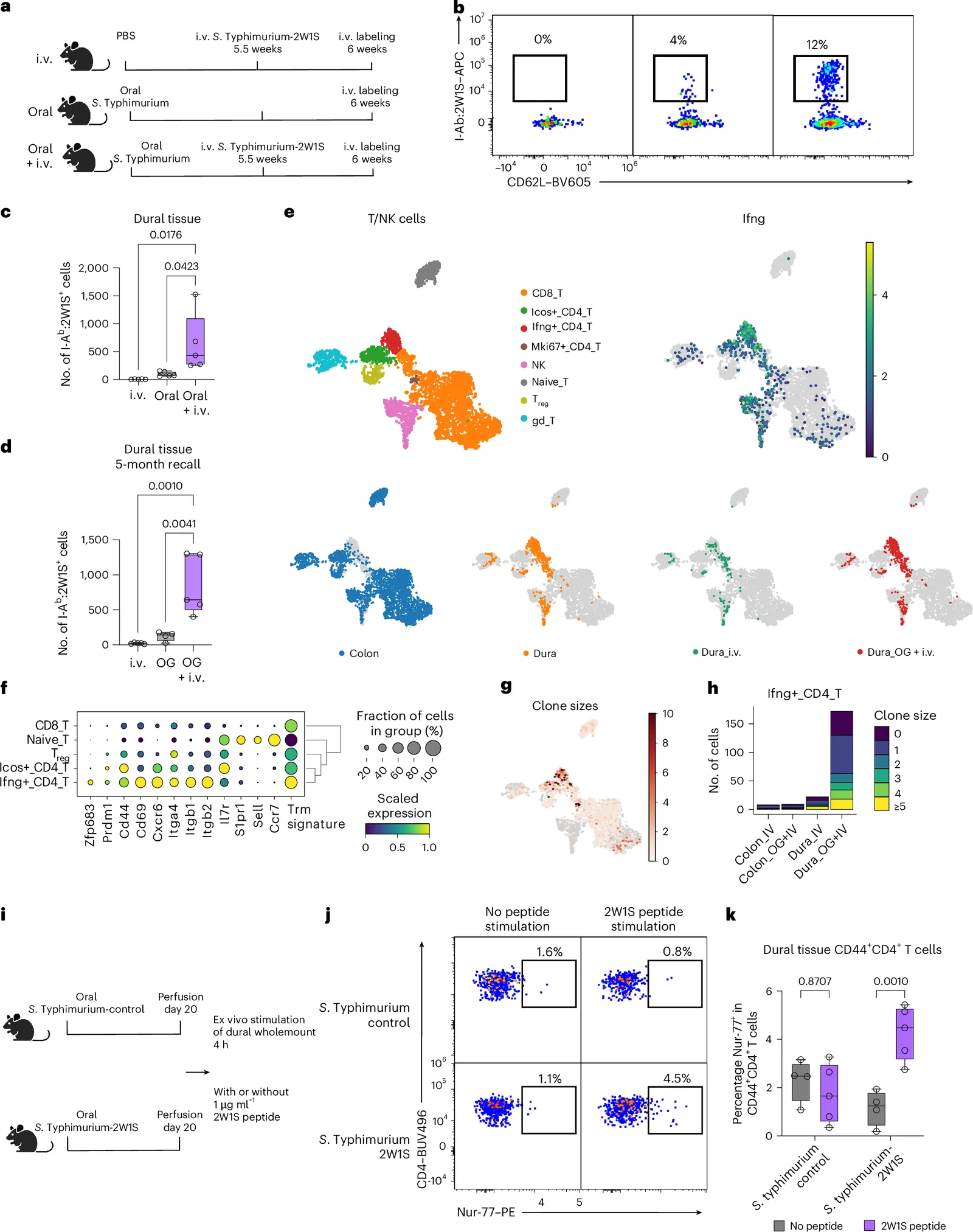
Intestinal challenges lead to long-lived dural antigen-specific memory CD4^+^ T cells. **a**, A schematic of experimental layout to detect memory expansion of dural antigen-specific CD4^+^ T cells. Mice were orally infected by gavage with *S*. Typhimurium-2W1S (‘Oral’ and ‘Oral + i.v.’) or PBS control (‘i.v.’) and 5.5 weeks the labeled groups (‘i.v.’ and ‘Oral + i.v.’) received an i.v. infection with *S*. Typhimurium-2W1S. 5 days post i.v., mice were culled with an anti-CD45 i.v. tail vein injection to identify memory expanded dural tissue- CD4^+^ 2W1S antigen-specific T cells. **b**,**c**, Representative flow cytometry plots (**b**) or cumulative data (**c**) showing the total number of dural extravascular CD4^+^ tetramer^+^ T cells between the different groups (*n* = 5 per group) as described in **a**. *P* values calculated using ordinary one-way ANOVA with Tukey’s multiple comparison test. **d**, Cumulative data showing total number of dural extravascular CD4^+^ tetramer^+^ T cells (i.v. group *n* = 5; oral gavage (OG) group *n* = 4; OG + i.v. group *n* = 5) in a 5-month recall model of the experimental layout in **a**. *P* values calculated using ordinary one-way ANOVA with Tukey’s multiple comparison test. **e**, The UMAP of 3,803 T and NK cells from scRNA-seq of extravascular (i.v. CD45^−^) CD45^+^ immune cells in the dura or colon, colored by cell type (left) or by tissue origin or infection arm (for dura only) (right). **f**, A dot plot of selected residency genes and enrichment of tissue-residency lymphocyte signatures in T and NK cells. **g**–**h**, Left: the UMAP of T and NK cells with paired scTCR-sequencing data colored by size of expanded T cell clones. Right: the bar plot of frequency of cells in the Ifng + _CD4_T cluster by condition, colored by clone size. **i**, A schematic of experimental layout to detect the activation of dural 2W1S-specific CD4^+^ T cells. Mice were orally infected with either WT *S*. Typhimurium or 2W1S-expressing *S*. Typhimurium. At 20 days post infection, the mice were culled with PBS perfusion. The dura was stimulated ex vivo with or without the presence of 2W1S peptide. **j**,**k**, Representative flow cytometry plots (**j**) and cumulative box plots (**k**) showing Nur-77^+^ dural tissue CD44^+^CD4^+^ T cells from the experiment described in **I** (*n* = 4 no peptide; *n* = 5 2W1S peptide group). *P* values calculated using two-way ANOVA with Bonferroni’s post test. In **c**, **d** and **k**, the box plot’s center line shows the median, the box shows the IQR and the whiskers spans from the maximum to minimum values. Illustrations in **a** and **i** created in BioRender; Neish, K. https://biorender.com/hv7riay (2026). Source data

Given that gut-polarized dural T cells were antigen-experienced and displayed an effector memory phenotype (CD62L^low^, CD44^high^), we wondered whether they were capable of expansion following rechallenge with an i.v. pathogen—consistent with our proposal that meningeal immunity is shaped by intestinal cues to protect the brain against gut-derived bacteria that enter the blood stream during barrier breach^32^. To test this, we challenged mice orally with 2w1s-*S*. Typhimurium and, 5.5 weeks later, rechallenged i.v. with the same pathogen (Fig. 5a). In mice receiving oral priming followed by an i.v. rechallenge, there was a significantly higher number of dural 2w1s-specific CD4^+^ T cells at day 5, compared with orally primed mice, whereas tetramer-positive cells were almost undetectable in mice receiving an i.v. challenge alone (Fig. 5b,c). Remarkably, these intestinal-derived dural CD4^+^ T cells were long-lived and capable of an antigen-specific recall response following i.v. rechallenge 5 months after the primary oral challenge (Fig. 5d).

**Extended Data Fig. 7:**
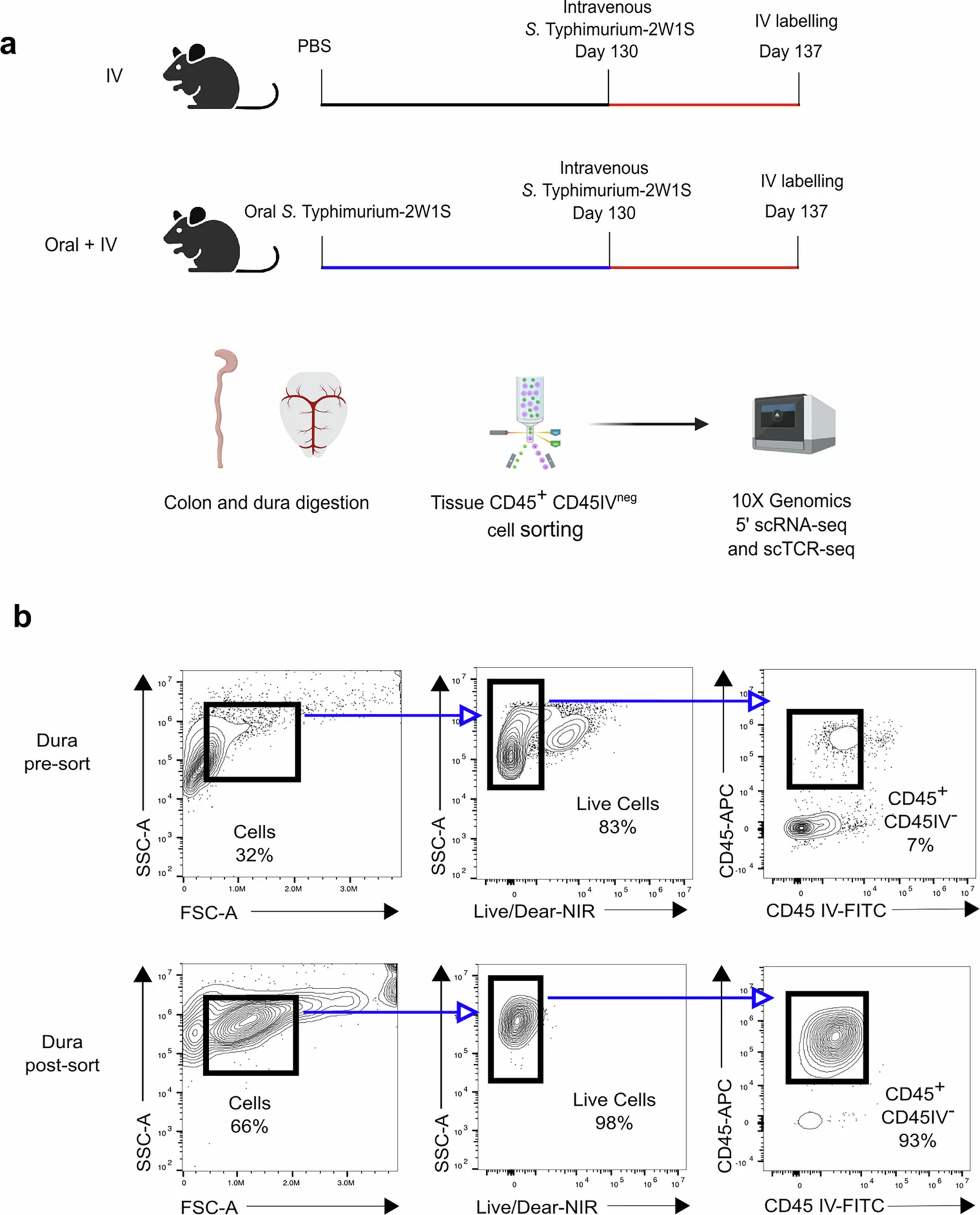
Gating strategies for FACS of dural extravascular CD45^+^ cells upon intestinal challenge. **A:** Experimental schematic for primary and recall intravenous *S*. Typhimurium infection for single-cell RNA sequencing and single-cell TCR sequencing. **B**: Pre-sort (top) and post-sort (bottom) flow plots from single cell sequencing experiments showing selection and enrichment for dural tissue (CD45IV^neg^) immune cells. Illustrations in **a** created in BioRender; Neish, K. https://biorender.com/2753atj (2026).

**Extended Data Fig. 8:**
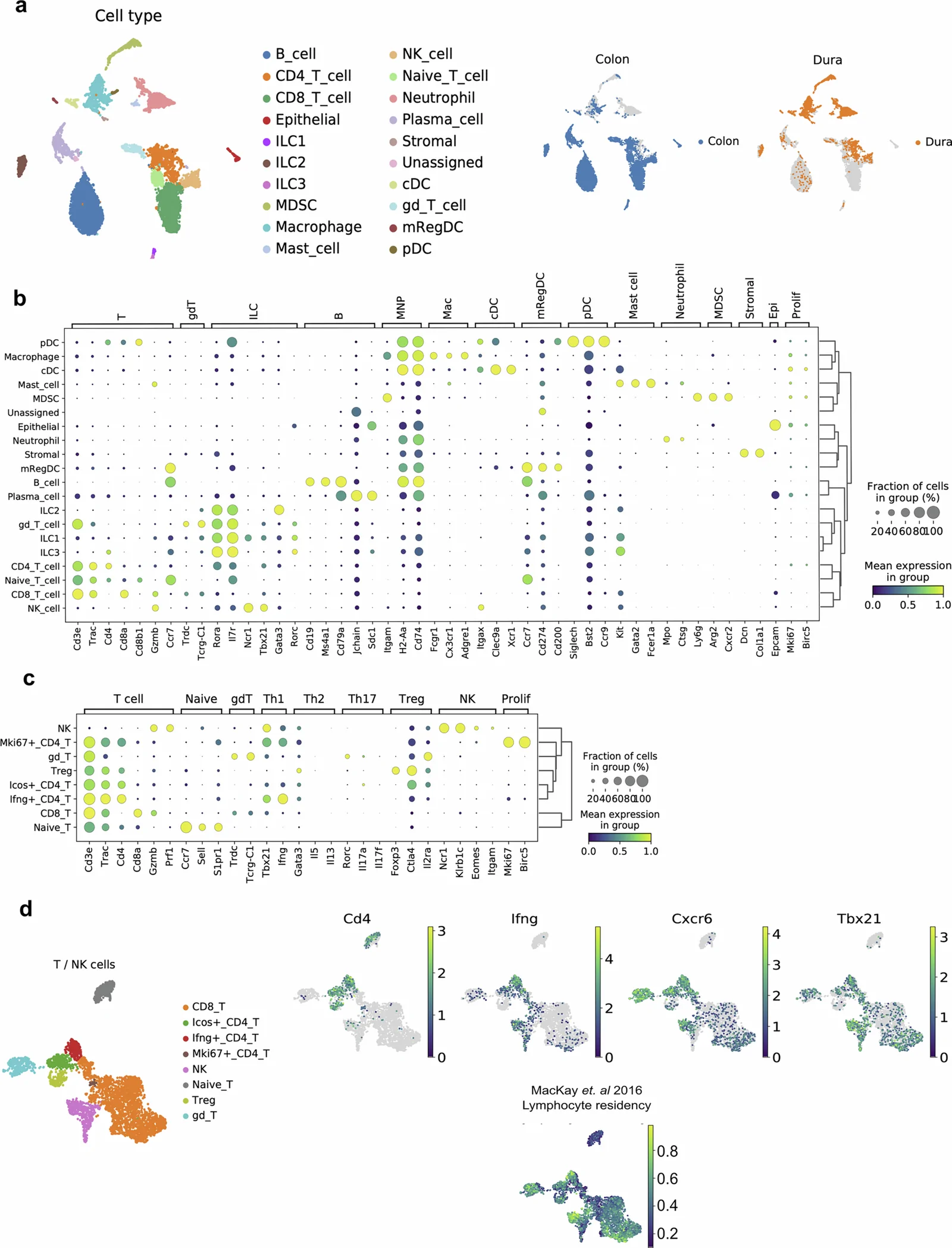
Annotated UMAP visualisation and gene expression of sequenced dural extravascular CD45+ cells. **A**: UMAP of 11655 cells obtained from scRNA-seq of extravascular CD45^+^ immune cells in the dura or colon, coloured by cell type and tissue origin. **B**: Dot plot of canonical markers gene expression of cell types in (**A**). **C**: Dot plot of canonical marker genes and selected differentially expressed genes of cell types in Fig. 5d. **D**: (left) UMAP of T and NK cells (subset from (a)) showing T and NK cell subsets and (right) UMAP of selected expressed CD4^+^ Th1 genes and lymphocyte residency markers from MacKay et. al (2016).

**Extended Data Fig. 9:**
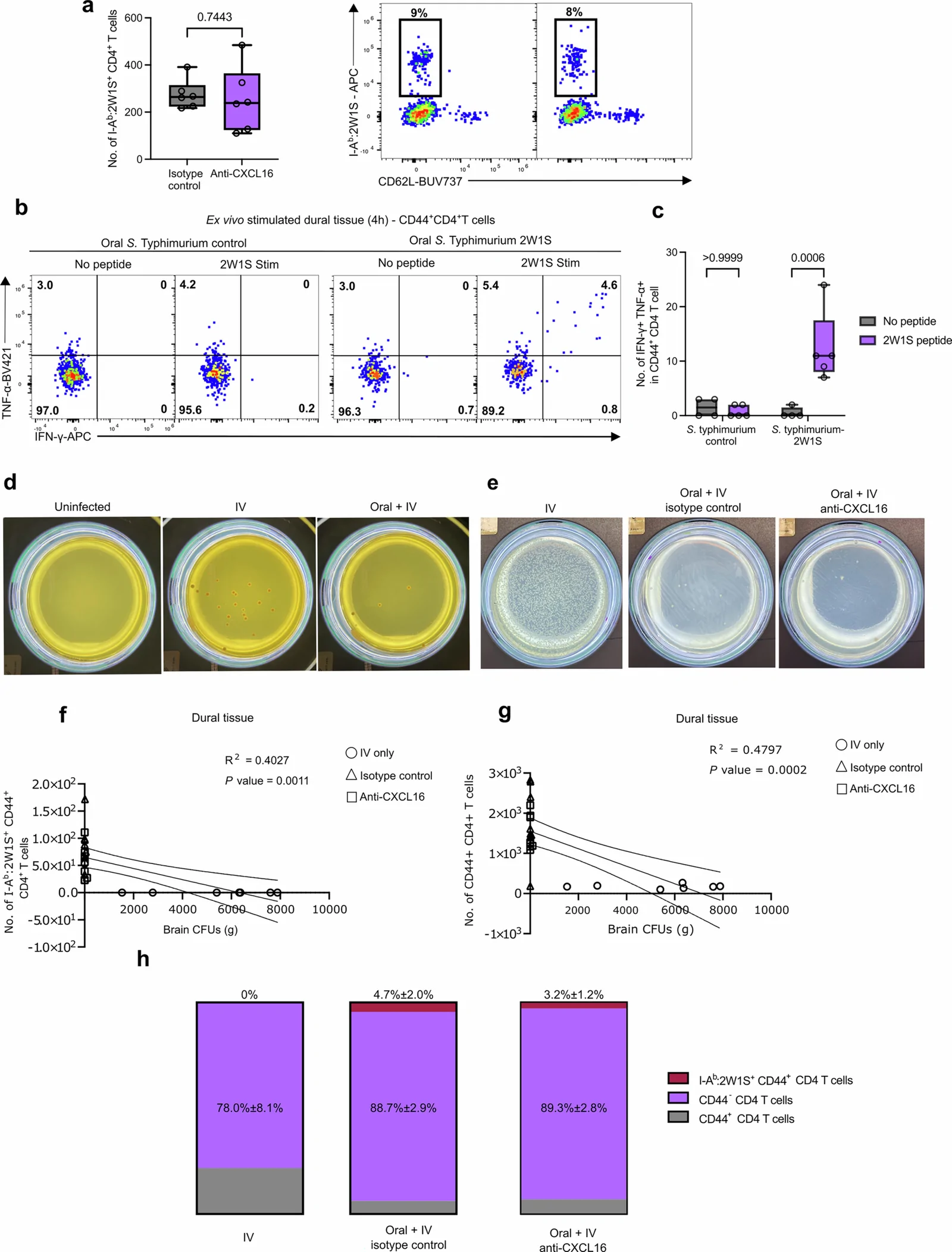
Dural antigen-specific memory CD4 T cells are capable of a recall response upon intravenous re-challenge. **A:** Cumulative plots (left) and representative flow cytometry plots (right) showing number of dural extravascular CD4^+^ tetramer^+^ T cells between mice infected with *S*. Typhimurium-2W1S followed by an intravenous re-call infection with *S*. Typhimurium*-*2W1S at day 28, with mice receiving either control isotype antibody or neutralising anti-CXCL16 antibody IP at day 28 and day 30 (*n* = 6 per group). *P* values calculated using two-tailed Student’s unpaired *t*-test (IV = intravenous, IP = intraperitoneal). **B-C:** Representative flow cytometry plots (left) and cumulative data (right) showing number of IFN-γ^+^ TNF-α^+^ CD44^+^ CD4^+^ T cells between the different groups (*n* = 4 no peptide group; *n* = 5 2W1S peptide group) as described in Fig. 5e. *P* values calculated using two-way ANOVA with Bonferroni’s post-test. **D:** Representative YPD plates of *C. albicans* growth in the different conditions as described in Fig. 5q. **E:** Representative agar plates of *S*. Typhimurium growth in the different conditions as described in Fig. 5k. **F:** Linear regression analysis with 95% confidence intervals (CI) plotting the relationship between brain CFUs per gram and the number of dural tetramer^+^ CD44^+^ CD4^+^ T cells in the conditions described in Fig. 5k. **G:** Linear regression analysis with 95% CI plotting the relationship between brain CFUs per gram and the number of dural CD44^+^ memory CD4^+^ T cells in the conditions described in Fig. 5k. **H:** Vertical slices showing percentage of tetramer^+^ CD44^+^ CD4 T cells, and percentage of CD44^+^ CD4 T cells among the total CD4 T cell population. Error bars represent SD. **A, C**: The box plots centre line shows the median, the box shows the IQR, and the whiskers spans from the maximum to minimum values. Source data

The scRNA-seq assessment of extravascular dural and colonic cells following oral priming with *S.* Typhimurium followed by i.v. challenge (oral + i.v.) or i.v. challenge alone (Extended Data Fig. 7a,b) confirmed the presence of an *Ifng*-expressing CD4^+^ T cell cluster, composed predominantly of cells derived from the dura of oral + i.v. animals (Fig. 5e and Extended Data Fig. 8a–c). These cells expressed memory and tissue-residency markers such as *Cd69* and showed enrichment of a published lymphocyte tissue-residency gene signature^33^ (Fig. 5f and Extended Data Fig. 8d). They also expressed integrin alpha 4 (*Itga4*) and beta 1 (*Itgb1*) that dimerize to form VLA4, previously shown to be expressed on human memory CD4^+^ T cells in cerebrospinal fluid^34^ and known to be important for lymphocyte entry into the CNS^35^. Expanded TCR clones were largely localized to this *Ifng*^+^CD4^+^ T cell population and mainly originated from the oral + i.v. group (Fig. 5g,h). To confirm that intestinally primed CD4^+^ T cells in the dura are capable of an antigen-specific recall response following i.v. challenge, independent of input from the circulating CD4^+^ T cell pool, we orally primed mice with 2w1s-*S*. Typhimurium and firstly confirmed that CXCL16 blockade during i.v. rechallenge with 2w1s-*S*. Typhimurium had no significant impact on dural CD4^+^ T cell expansion (Extended Data Fig. 9a). To definitively remove the possibility of circulating cell input into the dura, we orally challenged mice with 2w1s-*S*. Typhimurium BRD509 or control *S*. Typhimurium BRD509 and, at day 20, collected whole dura following vigorous i.v. perfusion to remove vascular contaminants. Dural wholemounts were then stimulated ex vivo with 2w1s peptide (Fig. 5i). At 4 h post stimulation, there was a significant increase in Nur-77^+^CD4^+^ T cells, indicative of antigen receptor signaling, as well as IFN-γ-expressing CD4^+^ T cells in the dura of oral 2w1s-*S*. Typhimurium*-*primed mice but not in the dura of mice primed with control *S*. Typhimurium BRD509 (Fig. 5j,k and Extended Data Fig. 9b,c).

### Oral priming induces antigen-specific CD4^+^ T cells in the dura and brain and is associated with protection from spread of i.v. bacteria into the brain

**Fig. 6:**
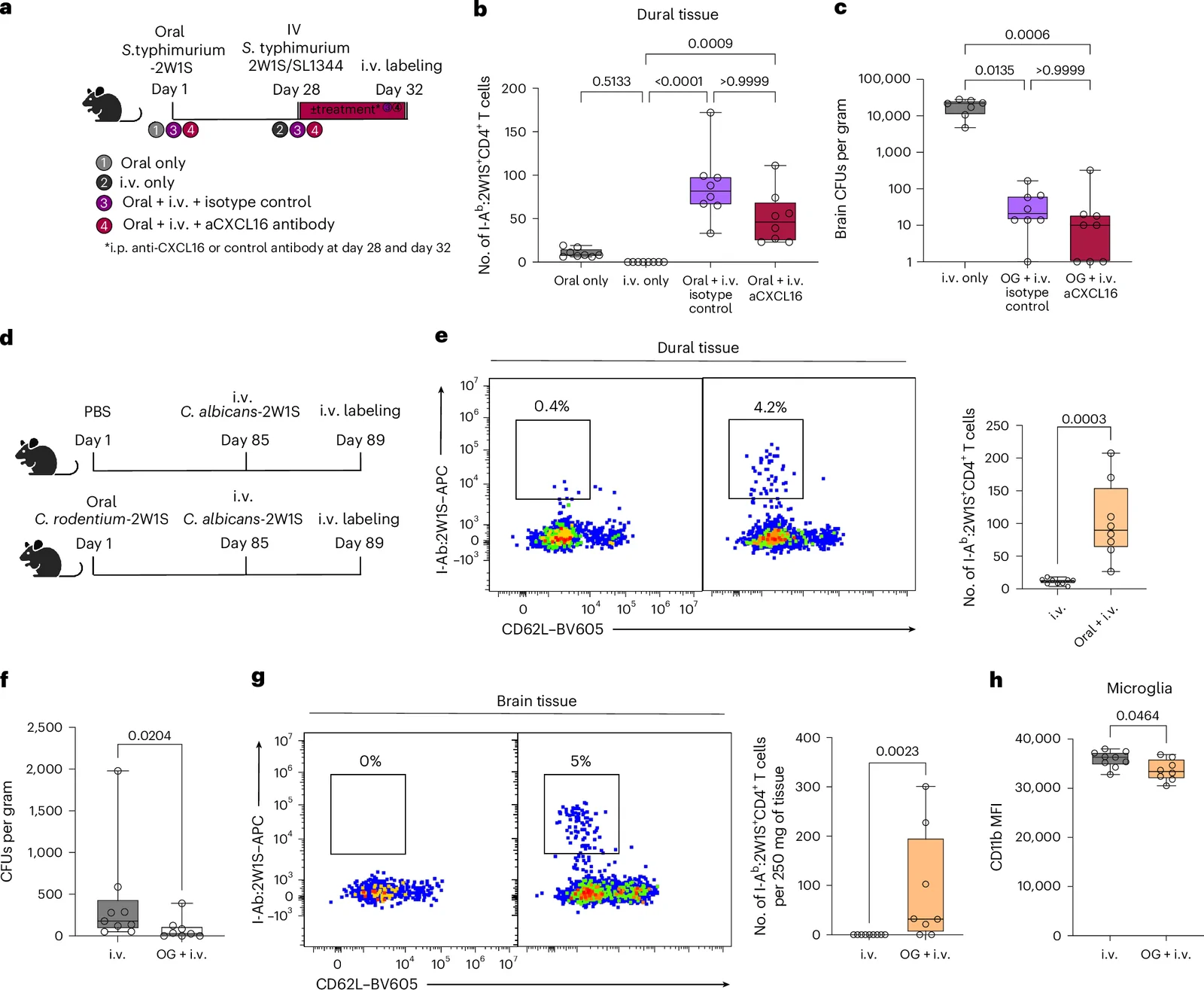
Dural and brain antigen-specific memory CD4^+^ T cells are capable of a recall response upon i.v. rechallenge. **a**, A schematic of experimental layout. Mice were given PBS control (‘i.v. only’) or infected by oral gavage with *S*. Typhimurium-2W1S (all other groups), followed by an i.v. recall infection with *S*. Typhimurium*-*2W1S and *S*. Typhimurium SL1344 strain at day 28 (apart from the ‘oral only’ group). Mice received either control isotype antibody (‘isotype control’) or neutralizing anti-CXCL16 antibody (‘aCXCL16 antibody’) intraperitoneally (i.p.) at the indicated time points. **b**, Cumulative box plots (*n* = 8 per group) showing percentage of dural extravascular CD4^+^CD44^+^ tetramer^+^ T cells between the groups in *P* values calculated using Kruskal–Wallis test with multiple comparison test. **c**, *S*. Typhimurium CFUs per gram of brain tissue between ‘i.v. only’, ‘oral + i.v. + isotype control’ and ‘oral + i.v. + aCXCL16 antibody’ groups as in **a** (*n* = 8 per group). Zero values were plotted as 1 for log scale visualization. *P* values calculated using Kruskal–Wallis test with multiple comparison test. **d**, A schematic of the experimental layout. Mice were given PBS control (top) (‘i.v.’) or infected by oral gavage with *C. rodentium*-2W1S (bottom) (‘oral + i.v.’); 85 days later, mice received an i.v. infection with *C. albicans*-2W1S and were culled 4 days later with an anti-CD45 i.v. tail vein injection. **e**, Representative flow cytometry plots (left) and cumulative box plots (*n* = 9 i.v. group; *n* = 8 oral + i.v. group) (right) showing dural extravascular CD4^+^ tetramer^+^ T cells between the groups in **d**. *P* values calculated using Student’s unpaired two-tailed *t*-test. **f**, *C. albicans* CFU per gram of brain tissue between the two groups in **d** (*n* = 9 i.v. group; *n* = 8 oral + i.v. group). *P* values calculated using a two-tailed Mann–Whitney *U* test. **g**, Representative flow cytometry plots (left) and cumulative box plots (*n* = 9 i.v. group; *n* = 8 oral + i.v. group) (right) showing brain tissue CD4^+^ tetramer^+^ T cells between the groups in **d**. *P* values calculated using Student’s unpaired two-tailed *t*-test. **h**, The cumulative box plot comparing mean fluorescence intensity (MFI) of CD11b between the two groups in **d** (*n* = 9 i.v. group; *n* = 8 oral + i.v. group). *P* values calculated using a Mann–Whitney *U* test. In **b**, **c** and **e**–**h**: the box plot’s center line shows the median, the box shows the IQR and the whiskers spans from the maximum to minimum values. Illustrations in **a** and **d** created in BioRender; Neish, K. https://biorender.com/hxple17 (2026). Source data

To test whether these dural memory CD4^+^ T cells contributed to brain defense, we infected mice orally with a 2w1s-expressing, attenuated *S*. Typhimurium BRD509 incapable of i.v. spread, followed by i.v. rechallenge with a 9:1 mixture of 2w1s-*S*. Typhimurium (to enable assessment of tetramer-positive cells) and a fully virulent WT *S*. Typhimurium SL1344 strain, capable of hematogenous spread into the CNS. We used an anti-Cxcl16 antibody to block the recruitment of orally primed CD4^+^ T cells into the dura from other sites during the recall phase (Fig. 6a). As we observed previously, i.v. rechallenge in mice with previous oral infection resulted in a robust increase in 2w1s-specific CD4^+^ T cells in the dura, with no significant difference between anti-Cxcl16- and isotype control-treated animals (Fig. 6b), confirming local, dural CD4^+^ T cell expansion, independent of new immigrants from the circulation. Importantly, mice receiving oral 2w1s-*S*. Typhimurium priming had a significant reduction in WT *S*. Typhimurium detectable in the brain, and this protection from brain spread was unaffected by Cxcl16 blockade during the i.v. rechallenge phase (Fig. 6c and Extended Data Fig. 9e). The number of dural 2w1s-specific cells showed a significant negative correlation with the number of brain colony-forming units (CFU), with an even stronger negative correlation with the number of CD44^+^ antigen-experienced CD4^+^ T cells (Extended Data Fig. 9f,g). Indeed, 2w1s-specific cells formed a small proportion of dural T cells, with a much larger proportional expansion in non-2w1s-specific CD44^+^CD4^+^ T cells evident in the orally primed group (Extended Data Fig. 9h), suggesting that oral priming with 2w1s-*S*. Typhimurium results in dural memory CD4^+^ T cells to multiple *S*. Typhimurium antigens beyond 2w1s in this model.

To specifically address the functional importance of dural CD4^+^ T cells, we attempted to deplete them using an anti-CD4 antibody administered in a hydrogel subcutaneously (s.c.) in the scalp. However, despite attempts to titrate down the dose, we were unable to avoid systemic CD4^+^ T cell depletion and target only dural CD4^+^ T cells. We therefore approached this question by using a cross-protection model that only involved a CD4^+^ T cell antigen. Specifically, we infected mice orally with 2w1s-*C. rodentium* and challenged them 80 days later with i.v. 2W1S-expressing *Candida albicans* (Fig. 6d), providing a model where the antigenic overlap between oral priming and i.v. recall is limited to a 14-amino acid CD4^+^ T cell epitope, 2w1s. This model therefore minimizes potential contributions to *Candida* defense from other cell types or antibodies and from memory CD4^+^ T cells recognizing non-2w1s antigens. At 4 days after i.v. candidal challenge, an expansion of dural extravascular 2W1S-specific CD4^+^ T cells was evident in oral 2w1s-*C. rodentium-*primed mice (Fig. 6e). This was associated with a reduced fungal load in the brain compared with mice challenged with i.v. *C. albicans* that had not undergone oral priming (Fig. 6f and Extended Data Fig. 9d), although this difference was modest compared with that observed in the *Salmonella* priming experiments. An expansion of 2W1S-specific CD4^+^ T cells was also evident on the extravascular brain parenchyma (Fig. 6g), in mice receiving oral priming with 2w1s-*C. rodentium*, with reduced expression of the activation marker CD11b also evident on microglia in orally primed mice (Fig. 6h). Altogether, these data suggest that gut-activated and gut-polarized, dural antigen-specific CD4^+^ T cells may make a contribution to CNS defense.

## Discussion

The dural vasculature is fenestrated, enabling bloodborne microorganisms to access the CNS. In humans, the commonest organisms observed in bacteremic subjects are gut-derived Gram-negative bacteria^36,37^. However, Gram-negative meningitis is infrequent^38–40^, suggesting that the dura has evolved effective defense mechanisms against gut-derived organisms^32^. We have previously shown that gut educated plasma cells localize to the walls of the dural venous sinuses, providing a defensive shield^4^. Here, we show that there is also an intimate link between gut and dural CD4^+^ T cells, where an infectious challenge in the gut not only activates and polarizes CD4^+^ T cells to ensure seeding of polarized, antigen-specific memory CD4^+^ T cells to the dura. Here, they reside, retaining the capacity to mount a recall response if the same antigen or pathogen is present in the circulation. This link between intestine and dura ensures that adaptive cellular immunity in the brain border is tailored to the most likely i.v. pathogen challenge. Although we attempted to specifically deplete CD4^+^ T cells in the dura using the local administration of a CD4 antibody in the scalp to test their relative importance to CNS defense, we were only able to achieve global, systemic CD4^+^ T cell depletion. However, our cross-priming experiments using a model where antigenic overlap was limited to a single CD4^+^ T cell peptide (2w1s) revealed a modest contribution in terms of reducing *Candida* spread to the brain in this stringent cross-protection model. We acknowledge that our inability to specifically deplete dural CD4^+^ T cells does not allow us to definitively prove a specific protective contribution from these cells.

Interestingly, we found antigen-specific gut-derived CD4^+^ T cells were also present in the extravascular space of several peripheral nonlymphoid organs (lung, liver, kidney and bladder). This is consistent with a recent study that tracked immune cells from the gut to peripheral organs by photoconverting intestinal cells in Kaede mice, identifying gut emigrants in the liver, lung and kidney and in a smaller number in the skin and bone marrow, with B cell the dominant migrants but CD4^+^ T cells also detected^41^. This suggests that immune cell seeding from the gut to other organs may be a more general strategy to ensure memory cells relevant to gut pathogens are present in nonlymphoid tissues to defend against hematogenous spread.

Previous studies have described the homeostatic movement of IFN-γ-expressing NK cells from the gut to the meninges, promoting the development of anti-inflammatory astrocytes^42^. In addition, CNS perturbations, such as ischemia, can lead to the induction of IL-17-producing T cells in the small intestine that traffic to the leptomeninges, with deleterious effects on infarct size^43^. Similarly, in experimental autoimmune encephalomyelitis, pathogenic intestinal-derived CXCR6-expressing T_H_17 cells were identified in the brain and spinal cord^44^, and a recent paper confirmed the movement of microbiome-specific gut CD4^+^ T_H_1 cells homeostatic traffic to the subfornical organ of the brain^45^. Here, we show that infectious challenges in the gastrointestinal tract can provide a cue to promote CD4^+^ T cell localization from the gut to the dura in a CXCR6–CXCL16-dependent manner, and we identify meningeal macrophages as the major source of CXCL16 in the dura. These dural CD4^+^ T cell were extravascular and expressed tissue-residency markers.

Meningeal T cells have the capacity to influence brain function via cytokine production. For example, CD4^+^ T cell-derived IFN-γ can influence social behavior^10^, T cell-specific IL-4-deficiency leads to deficits in memory and cognition^11^ and γδ T cell-derived IL-17 promotes synaptic plasticity and short term memory^12^ but in excess can increase anxiety-like behavior owing to direct effects on IL-17 receptor-expressing cortical glutamatergic neurons^13^. A number of studies have also shown that the gut microbiome can influence behavior and susceptibility to anxiety and depression, including via IL-17^14–17^. The assumption had been that these effects on brain function were due to the impact of increased circulating cytokines produced by T_H_17 cells in the gut^17^. Our data provide an alternative explanation, showing that cytokine-producing CD4^+^ T cells activated by gut microorganisms may localize to the dura. We also found that during DSS colitis, a model of IBD, there was an expansion of T_H_17 cells in the dura. This observation has important implications for the mechanistic underpinning of the increase in mental health problems described in patients with IBD, with up to 30% and 25% of patients experiencing anxiety and depression symptoms, respectively^46^. Our work raises the possibility that gut-derived IL-17-secreting CD4^+^ T cells may contribute to this phenomenon, particularly given the recent observation of arachnoid cuff entry points that directly link the dura with the leptomeninges^47^, providing a channel through which CD4^+^ T cell-derived cytokines may traffic to the brain.

Schistosomiasis affects 140 million people worldwide and is prevalent in poor communities without clean water in tropical and subtropical areas, particularly in sub-Saharan Africa^48^. Here, we show that *S. mansoni* infection is associated with a substantial expansion in dural T_H_2 cells, with important clinical implications. Although IL-4 deficiency has been associated with deficits in memory and learning, the effect of a chronic excess of IL-4 in the brain has not been assessed and could contribute to the epidemiological associations observed between chronic schistosomiasis and academic attainment that were first described more than 70 years ago^49,50^.

More broadly, our work has implications for our understanding of how the brain might sense systemic perturbations. We have long known that sensory neurons provide a direct means by which information from peripheral organs, including the gut, can be conveyed to the brain. Our findings here that CD4^+^ T cells, and previously, B and plasma cells^8^, can migrate to the dura provide an additional mechanism by which information can be transmitted to the CNS, utilizing the physical relocation of immune cells that communicate with neurons via cytokines, with cytokine–receptor expression increasingly recognized on cells of the CNS^10,51–54^. This provides an information relay system distinct from neuronal signaling, using migratory lymphocytes rather than axons that physically span the distance between peripheral organs and the brain and cytokines rather than neurotransmitters to transfer signals from cell to cell. Ultimately, this link between intestinal and dural immune cells allows the CNS to accrue immunological memory of microorganisms most likely to enter the blood and compromise organismal survival.

In summary, our data shows that gut challenge establishes and shapes long-lived CD4 memory in the dura capable of rapid recall in response to bloodborne pathogens, with the potential to influence brain function and behavior via local cytokine production.

## Methods

### Mice

Mice experiments were carried out in a specific pathogen-free conditions at the University of Cambridge and The Wellcome Trust Sanger Institute where specified under relevant project licenses with institutional study plan approval by a local animal welfare and ethical review board and in accordance with the United Kingdom Animals (Scientific Procedures) Act of 1986. Mice were maintained in standard 12 h light and 12 h dark cycle. Humidity was maintained between 45% and 65% and temperature set at 21 ± 2 °C. Basic husbandry and animal welfare was provided daily by trained and competent animal technicians.

WT mice were on a C57BL/6J background unless otherwise specified. WT mice were either bred in house or purchased from Jackson Laboratories. Where mice were purchased from a commercial vendor, they were allowed to acclimatize for at least a week before the commencement of the experiment. For all animal experiments, mice aged between 6 and 20 weeks were used unless otherwise stated. Female mice were used for all WT mice experiments. For other genotypes and for experiments involving the generation of bone marrow chimeras, both female and male mice were used. For. *S. mansoni* infections, TO outbred (HsdOla:TO) or BALB/C mice were used (for flow cytometry or imaging, respectively) under the license of Gabriel Rinaldi (P77E8A062). RORγt–eGFP reporter mice were kindly provided by Andrew McKenzie (MRC-LMB) and maintained in house for several generations.

Mice were given ad libitum access to food (Safe 105, Safe Diets) and water unless otherwise specified. Following i.v. infections with *S.* Typhimurium or *C. albicans*, mice were additionally given Pure-Water Gel: Total Hydration (PWG-2, Bio-Serv) and a powdered version of the above food mixed with water in Petri dishes on their cage floor daily.

At 5 min before death, mice were given i.v. anti-CD45-labeling antibody (1.5 μg per mouse in 100 μl sterile PBS) where appropriate. Mice were killed by rising CO_2_ exposure followed by either cervical dislocation or perfusion via the heart with ice-cold PBS to confirm death and relevant organs dissected. Peripheral blood was taken to confirm successful blood CD45 labeling.

### Bone marrow chimera

CD45.1^+^/CD45.2^+^ host mice were irradiated with a split dose on the same day (2 × 5.5 Gy) followed by immediate i.v. reconstitution with sex-matched bone marrow cells (~4 × 10^6^ cells) composed of a 50:50 mix of CD45.1+ WT:CD45.2 CXCR6^gfp/gfp^ genotypes. At 6 weeks following irradiation mice were orally infected with *S.* Typhimurium-2W1S as described in ‘*S*. Typhimurium oral and i.v. infections’ section below. At 3 weeks following oral infection, mice received a CD45-i.v. injection 5 min before killing, and organs were taken for flow analysis.

### *S*. Typhimurium oral and i.v. infections

For in vivo infections, frozen glycerol (20% v/v) stocks of *S*. Typhimurium BRD509 strain, 2W1S-expressing strain SPN555^55^ or SL1344 strain were defrosted at room temperature (RT) and 20 μl streaked on an agar plate. Plates were incubated upside down overnight at 37 °C. The following day, three to four individual colonies were gently taken up with the end of a sterile stripette and added to 100 ml LB broth in a 250-ml conical flask and incubated at 37 °C overnight with no shaking. The following day, the culture was diluted 1 in 10 in sterile PBS for oral infections to create inoculums of 1 × 10^7^ CFUs per 200 μl. For i.v. infections the culture was diluted 1 in 50 to create inoculums of 1 × 10^6^ CFUs per 100 μl. For mixed i.v. infections, the 2W1S strain was diluted as above to 1 × 10^7^ CFUs per milliliter while the SL1344 strain was diluted to 5 × 10^4^ CFUs per milliliter. The two cultures were mixed at a 9:1 ratio (2W1S:SL1344) to create inoculums of roughly 1 × 10^6^ CFUs of 2W1S and 500 CFUs of SL1344 per mouse. For all infections, inoculums were plated immediately post infection on agar plates containing 100 μg ml^−1^ streptomycin at 37 °C overnight to confirm infection CFU counts. For oral infections, mice were gavaged 24 h before infection with 20 mg of streptomycin (SB0494, Bio Basic) in 100 μl sterile PBS to aid colonization. For oral infections, fecal CFUs were plated 4–10 days post oral gavage to confirm the stable colonization of mice.

For enumeration of *S*. typhimurium CFUs in brain tissue, homogenates were spread on agar plates containing 100 μg ml^−1^ streptomycin. Following dissection from the skull, brains were weighed and added to 500 μl sterile PBS in a 5-ml bijou tube. Brains were then homogenized using a tissue homogenizer. The homogenate was briefly (~5 s) spun down in a desktop centrifuge to remove large debris, and 400 μl of homogenate was added to an agar plate and spread using glass beads. Plates were then incubated upside down at 37 °C, 5% CO_2_ for 24 h and visible colonies counted. CFUs were calculated per gram of brain tissue per mouse.

### *C. rodentium* oral infections

For in vivo *C. rodentium* infections, frozen 20% glycerol (v/v) stocks of WT ICC180 strain, *espO* deletion mutant ICC1333 strain^56^ or *C. rodentium*-expressing 2W1S strain were defrosted at RT and 10 μl streaked on an agar plate. Plates were then incubated upside down overnight at 37 °C. The following day, three to four individual colonies were gently taken up with the end of a sterile stripette and added to 100 ml LB broth in a 250-ml conical flask and incubated at 37 °C overnight with shaking (180 rpm). The following day, the culture was spun down and concentrated 10× in sterile PBS to create an approximately 1 × 10^9^ CFUs per 200 μl inoculum for oral gavage. For all infections, inoculums were plated immediately post infection on agar plates at 37 °C overnight to confirm infection CFU counts. For oral infections, fecal CFUs were plated 4–10 days post oral gavage on to confirm stable colonization of mice.

### Construction of *C. rodentium* expressing 2W1S

The 2W1S peptide was inserted between positions 126 and 127 of the *C. rodentium* EspA translocator protein in strain ICC169. The homology regions flanking the insertion site of *espA*, comprising the upstream and downstream 300 bp, and the intertwine sequence of the peptide, were synthesized (Thermo) and cloned into the SacI-SphI sites of pSEVA612S^57^. The resulting pSEVA612S-2WtagEspA was transformed into *Escherichia coli* CC118λpir^58^. The endogenous *C. rodentium espA* gene was then replaced with 2WtagEspA via triparental conjugation as described^59^. All the plasmids and mutants were confirmed by sequencing (Eurofins).

### *S. mansoni* life cycle maintenance and infections

The life cycle of the NMRI (Puerto Rican) strain of *S. mansoni* was maintained by infection of TO outbred (HsdOla:TO) female mice and *Biomphlaria glabrata* snails. Cercariae were collected from infected snails by exposure to bright light and the number of live cercariae counted under a dissecting microscope. Mice were infected percutaneously via the tail vein with 300 cercariae over the course of 40 min. To accomplish this, mice were restrained in a bespoke isoflourane anesthesia rig inside a restrainer and their tail veins inserted into a test tube containing 500 μl of a stock solution of 600 cercariae per milliliter. Mice were killed 42 days after challenge by intraperitoneal (i.p.) injection of 200 mg ml^−1^ pentobarbital supplemented with 100 U ml^−1^ heparin. Mice were intracadially perfused with phenol red-free DMEM supplemented with 10 U ml^−1^ heparin and liver tissues collected and digested to isolate eggs to maintain the life cycle infection of the snail. Organs were collected processed as described.

### *T. muris* infections

Mice were infected by oral gavage with a low (20–25) dose of embryonated eggs from *T. muris* E-isolate. Mice were killed at day 21 with an anti-CD45 i.v. label and relevant organs dissected.

### *C. albicans* i.v. infections

For in vivo infections, frozen glycerol (20%, v/v) stocks of recombinant *C. albicans* expressing 2W1S^60^ were defrosted at RT and 20 μl streaked on a yeast extract peptone dextrose (YPD) plate (2% peptone, 2% glucose, 1% yeast extract, 2% agar, 1% penicillin–streptomycin). The YPD plate was then incubated upside down overnight at 30 °C, 5% CO_2_. The following day, three to four individual colonies were gently taken up with the end of a sterile stripette and added to 50 ml YPD broth (2% peptone, 2% glucose, 1% yeast extract) in a 250-ml conical flask and shaken at 180 rpm for 18 h at 30 °C, 5% CO_2_. *C. albicans* cells were then collected by spinning for 4 min at 500*g* and washed twice in sterile PBS. Cells were enumerated under a light microscope to create the desired inoculum concentration as specified in the relevant experiments, and all i.v. injections were in 100 μl sterile PBS. For infection experiments with *C. albicans*, inoculums were plated immediately post infection on YPD plates at 30 °C overnight to confirm exact infection CFU counts.

For the enumeration of *C. albicans* CFUs in brain tissue, homogenates were spread on YPD plates, similar to that described above. Following dissection from the skull, brains were sliced in half using a scalpel. One half was weighed and added to 500 μl sterile PBS in a 2-ml Eppendorf tube. Brains were then homogenized by firmly drawing them in and out of 1-ml plastic syringe several times with force to create a crude homogenate. Homogenate was briefly (5 s) spun down in a desktop centrifuge to remove large debris, and 100 μl of homogenate was added to a YPD plate and spread using glass beads. Plates were then incubated upside down at 30 °C, 5% CO_2_ for 48 h and visible colonies counted. CFUs were calculated per gram of brain tissue per mouse.

### CXCL16 neutralization

Mice were infected with *S*. Typhimurium-2W1S as described above. On day 5 post infection, mice received an s.c. injection of 200 μl of anti-CXCL16 antibody (1 mg ml^−1^, MAB503, BioTechne) or Rat IgG2a control antibody (1 mg ml^−1^, MAB006, BioTechne) transcranially on the top of the head in a carboxymethylcellulose hydrogel (10 mg ml^−1^ in sterile PBS, C9481, Sigma-Aldrich) under light isoflurane anesthesia. Mice then subsequently received i.p. injections of the specified antibodies at the concentrations above in 100 μl sterile PBS every second day until day 17. Mice were killed on day 20 with a CD45-i.v. injection 5 min before death. Treatment groups were equally split between cages to minimize cage effects.

### CXCL16 neutralization following i.v. rechallenge

Mice were orally infected with *S.* Typhimurium-2W1S and i.v. rechallenged at day 28 as described above. On the day of rechallenge and at day 30, mice received 100 μl of anti-CXCL16 antibody (1 mg ml^−1^, MAB503, BioTechne) or Rat IgG2a control antibody (1 mg ml^−1^, MAB006, BioTechne) i.p. injections to block CD4^+^ T cell recruitment from the circulation. Mice were killed at day 32 with a CD45-i.v. injection 5 min before death.

### VLA-4 and LFA-1 neutralization

Mice were infected with *S*. Typhimurium-2W1S as described above. On the day of oral infection, mice received a s.c. injection of 100 μl of anti-VLA-4 (2 mg ml^−1^, BE0071, Bio X Cell) and anti-LFA-1a (2 mg ml^−1^, BE0005, Bio X Cell) or Rat IgG2b control antibody (2 mg ml^−1^, Bio X Cell, BE0090) transcranially on the top of the head in a carboxymethylcellulose hydrogel (10 mg ml^−1^ in sterile PBS, C9481, Sigma-Aldrich) under light isoflurane anesthesia. Mice then subsequently received i.p. injections of the specified antibodies at the concentrations above in 100 μl sterile PBS every second day from day 3 until day 23. Mice were i.v. rechallenged at day 25 and killed on day 29 with a CD45-i.v. injection 5 min before death.

### DSS-induced colitis

To induce colitis for flow cytometry experiments, mice were given 2.5% (w/v) DSS (MP Biomedicals) in drinking water for 5 days, followed by 14 days on normal drinking water, a further 5 days on 2.5% DSS and a final 6 days on normal drinking water before killing.

To induce colitis for TCR-sequencing experiments, mice were given 2.5% (w/v) DSS in drinking water for 7 days, followed by 14 days on normal drinking water before killing.

### Colon tissue processing

The colon was removed immediately following death by cutting the pubic bone and removing the colon from rectum to where it joins the cecum. Extraneous fat was removed using a curved scissors and fecal content gently extruded using a forceps. The colon was then threaded on to scissors, cut open longitudinally and washed in 30 ml ice-cold PBS–HEPES in a Petri dish. Colon tissue was then cut into 0.5-cm pieces, placed in epithelial stripping buffer in a bijou tube (5 ml RPMI, 2% FCS, 10 mM HEPES, 5 mM EDTA, 1 mM dithiothreitol (DTT)) at 37 °C shaking for 25 min. Post incubation, tubes were vortexed vigorously to release epithelial cells and the solution passed through a wire-mesh filter to retain the epithelial-stripped tissue and discard epithelial cells. Tissues were washed in 30 ml PBS–HEPES to remove excess DTT and then added to digest buffer in a bijou tube (5 ml RPMI, 2% FCS, 10 mM HEPES, 1 mg ml^−1^ collagenase I (17100017, Thermo Fisher Scientific), 100 μg ml^−1^ DNAse I (10104159001, Merck)). Digestion was carried out with shaking at 37 °C for 30–40 min. Post digestion, tubes were vortexed vigorously to aid tissue breakdown and lamina propria cell release. Homogenate was then passed through a 70-μm cell filter, tissue mashed using the rubber end of a 1-ml syringe insert and the filter washed through with 10 ml 3% FCS PBS. Cells were spun down (500*g*, 10 min) and resuspended in 5 ml 40% Percoll solution. This solution was then layered on top of an 80% Percoll solution and centrifuged at 600*g*, 20 min, with no brake and no acceleration. Enriched immune cells were then collected at the 80/40 interface using a 1-ml pipette and washed in 10 ml 3% PBS–FCS (500*g*, 5 min), and samples were moved to a 96 V-well bottom plate for flow cytometry staining.

### Dura tissue processing

Skull cap dura was dissected in ice-cold PBS under a dissecting microscope using dissection scissors and fine anti-static tweezers. For dural digestion to obtain a single-cell suspension, dura was placed in 1 ml digest medium (1 ml RPMI, 2% FCS, 10 mM HEPES, 2.5 mg ml^−1^ collagenase D (11088866001, Thermo Fisher Scientific), 125 μg ml^−1^ DNAse I (10104159001, Merck)) post-dissection at 37 °C for 30 min. Following incubation, dura was vortexed vigorously to aid cell release and tissue breakdown and firmly pipetted up and down several times. The sample was then passed through a 70-μm FACS tube filter, washed and moved to a 96 V-well bottom plate for flow cytometry staining.

### Spleen tissue processing

Spleen was removed immediately following death and extraneous fat removed with scissors, placed on a 70-μm cell filter and mashed using the rubber end of a 1-ml syringe insert. Cells were washed with 3% PBS–FCS, spun down and resuspended in 1 ml RBC lysis buffer for 1 min. Lysis was quenched by adding excess 3% FCS PBS and cells were spun. Supernatant was discarded and samples were moved to a 96 V-well bottom plate for flow cytometry staining.

### Ex vivo stimulation of cells for cytokine and chemokine and transcription factor detection

For the detection of cell-specific cytokine production by flow cytometry, cells were stimulated ex vivo following tissue digestion, except for dural tissue, which was stimulated after dissection as a whole mount from the skullcap. Following stimulation, the dura was then digested as described. It was found that this substantially improved the viability of the dural immune cells rather than stimulation as a single-cell solution. For cytokine analysis, cells from the relevant organs were cultured for 4 h at 37 °C, 5% CO_2_ in complete RPMI (RPMI plus 10% FCS and 10 mM HEPES) plus phorbol 12-myristate 13-acetete (PMA) (20 ng ml^−1^, Sigma-Aldrich, P1389), ionomycin (1 μg ml^−1^, Sigma-Aldrich, I9657) and BD GolgiPlug protein transport inhibitor (1:1,000, 555029, BD Biosciences) or Monensin (1:1,000, eBioscience, 00-4505-51). For chemokine analysis, cells were stimulated as above ex vivo but without the presence of PMA or ionomycin. For the detection of cytokine production and transcription factor expression of antigen-specific cells, cells were stimulated as above but with the presence of 2W1S peptide (peptide sequence EAWGALANWAVDSA, 1 μg ml^−1^, GenScript) instead of PMA–ionomycin. Following incubation, cells were washed twice in ice-cold PBS and transferred to a 96-well V-bottom plate for downstream flow cytometry staining as described.

### Flow cytometry staining

Staining was carried out in 96-well V-bottom plates (Thermo Scientific Fisher, 612V96). All spins in plates were carried out at 800*g*, 2 min. Before extracellular staining, cell suspensions were blocked against nonspecific antibody staining in 50 μl 3% PBS–FCS containing 0.5% (v/v) heat-inactivated normal mouse/rat serum (Invitrogen, 10410/Thermo Scientific Fisher, 10-710-C). For tetramer staining, cells were incubated with tetramers in PBS (1:400) at RT for 1 h. Tetramers were mouse I-A(b) MHC molecules loaded with 2W1S peptide sequence (EAWGALANWAVDSA) conjugated to either allophycocyanin (APC) or phycoerythrin (PE) as specified in the relevant texts and figures. Tetramers were produced and provided by the NIH Tetramer Facility. Following blocking, cell suspensions were incubated with antibodies in 3% PBS–FCS at the dilutions specified below on ice for 45 min, followed by two washes in 3% PBS–FCS. To discriminate live from dead cells, cell suspensions were stained with ViaKrome 808 Fixable Viability Dye (Beckmann Coulter, C36628) at 1:250 in PBS at RT for 20 min or with Live/Dead Near-IR (Invitrogen, 1:500). If no further intracytoplasmic or intranuclear staining was performed, cells were then fixed for 4 min at RT in 4% paraformaldehyde and washed in 3% PBS–FCS before staining. For cytokine or chemokine detection, cells were fixed and permeabilized in 200 μl CytoFix/CytoPerm (BD Biosciences, 554714) for 40 min at RT, washed twice using the supplied Perm/Wash buffer and resuspended in intracellular anti-cytokine antibodies in Perm/Wash buffer for 30 min at RT at dilutions specified below. For intracellular antibody staining, cell suspensions were fixed and permeabilized with 100 μl of the fixation buffer in the Foxp3 Transcription Factor Staining Kit (eBioscience, 11500597) for 45 min at RT, followed by two washes in the supplied Perm Buffer. Cell suspensions were then resuspended in antibodies in Perm Buffer for 30 min at RT at dilutions specified below, washed two further times in supplied Perm Buffer and resuspended in 3% PBS–FCS for flow cytometry analysis. Flow cytometry was performed on either a CytoFLEX LX Flow Cytometer (Beckman Coulter), a ID7000 Spectral Cell Analyzer (Sony) at the Core Flow Facility at the Laboratory of Molecular Biology or on a Cytek Aurora Spectral flow cytometer (Cytek Biosciences) at the NIHR Cambridge BRC Cell Phenotyping Hub. Cell sorting was performed on a BD Influx (BD Biosciences).

Antibodies for cell staining are as follows (clone, cat. no., supplier, dilution): anti-CD45 BUV395 (30 F-11, 564279, BD Biosciences, 1:200), CD45.1 BUV395 (A20, 565212, BD Biosciences, 1:200), anti-CD4 BUV496 (GK1.5, 612952, BD Biosciences, 1:200), anti-PD-1 BUV615 (RMP1-30, 752354, BD Biosciences, 1:200), anti-CD19 BUV661 (1D3, 612971, BD Biosciences, 1:200), anti-CD62L BUV737 (MEL-14, 612833, BD Biosciences, 1:200), anti-CD8α BUV805 (53-6.7, 612898, BD Biosciences, 1:200), anti-TCRβ BV421 (H57-597, 109230, BioLegend, 1:100), anti-TNF-α BV421 (MP6-XT22, 506328, BioLegend, 1:200), anti-anti-MHC-II eFlour450 (AF6-120.1, 48-5320-82 eBioscience, 1:400), anti-GATA-3, eFluor450 (TWAJ, 48-9966-42, eBioscience, 1:200), anti-CD45 PacBlue (30-F11, 103126, BioLegend, 1:200), anti-TCRβ BV510 (H57-597, 563221, BioLegend, 1:100), anti-CD90.2 BV510 (53-2.1, 140319, BioLegend, 1:400), anti-CD3ε BV605 (145-2C11, 100351 BioLegend, 1:100), anti-CD62L BV605 (MEL-14, 104437 BioLegend, 1:200), anti-CD11b BV650 (M1/70, 101239, BioLegend, 1:300), anti-CD62L BV786 (MEL-14, BioLegend, 564109), anti-Gr1 BV786 (RB6-8C5, 740850 BD Biosciences, 1:400), anti-Ki-67 AF488 (SolA15, 53-5698-82, eBioscience, 1:200), anti-IFN-γ-FITC (XMG1.2, 505806 BioLegend, 1:200), anti-CD45 FITC (30-F11, 103108 BioLegend, 1:200), anti-CD11c FITC (N418, 35-0114, eBioscience, 1:200), anti-CD11b FITC (M1/70, 101206, BioLegend, 1:300), anti-CD3 SparkBlue574 (17A2, 76530-944, VWR, 1:200), anti-CD44 PerCP (IM7, 103036, BioLegend, 1:100), anti-CD19 PerCP (6D5, 115532 BioLegend, 1:200), anti-B220 PerCP-Cy5.5 (RA3-6B2, 103236 BD Biosciences, 1:200), anti-GATA-3 PE (TWAJ, 12-9966-42, eBioscience, 1:200), anti-IL-17a PE (eBio17B7, 12-7177-81 eBioscience, 1:200), anti-T-bet PE (eBio4B10, 12-5825-82, eBioscience, 1:200), anti-CXCL16 PE (12-81, 566740 BD Biosciences, 1:200), anti-CXCR6 PE (SA051D1, 151103 BioLegend, 1:200), anti-Nur-77 PE (12.14, 12-5965-82, eBioscience, 1:200), anti-CD69 PE/Dazzle594 (H1.2F3, 104536, BioLegend, 1:200), anti-CD73 PE/Dazzle594 (TY/11.8, 127234, BioLegend, 1:200), anti-RORγt PE-Cy7 (B2D, 25-6981-82, eBioscience, 1:200), anti-TCRγδ PE-Cy7 (GL3, 118124, BioLegend, 1:200), anti-Podoplanin PE-Cy7 (8.1.1, 127411, BioLegend, 1:200), anti-CD62L PE-Cy7 (MEL-14, 104418, BioLegend, 1:200), anti-CD45.2 PE-Cy7 (104, 25-0454-82, eBioscience, 1:200), anti-FR4 AF647 (12A5, 125009, BioLegend, 1:200), anti-RORγt APC (B2D, 17-6981-82 eBioscience, 1:200), anti-GATA-3 APC (TWAJ, 50-9966-42 eBioscience, 1:200), anti-CD64 APC (X54-5/7.1, 139306, BioLegend, 1:200), anti-CD45 APC (QA17A26, 157605, BioLegend, 1:200), anti-IFN-γ APC (XMG1.2, 554413, BD Biosciences, 1:200), anti-GATA-3 eFluor660 (TWAJ, eBioscience, 50-9966-42, 1:200), anti-FoxP3 AF700 (FJK-16s, 56-5773-82, eBioscience, 1:200), anti-CD8 APC-Cy7 (YTS156.7.7, 126620, BioLegend, 1:200), anti-CXCR6 APC-Cy7 (SA051D1, 151123, BioLegend, 1:200) and anti-CD45 APC-eFluor780 (30-F11, 47-0451-82, Invitrogen, 1:200).

### Confocal imaging and staining

For imaging studies, mice were intracardially perfused with 20 ml ice-cold PBS and skull caps fixed in Antigenfix (P0016, Diapath) for 90 min on ice, followed by washing in PBS. Dura was blocked at RT for 30 min in blocking–permeabilization solution containing 2% normal rat serum, 2% normal mouse serum, 0.1% Triton X-100, 0.05% Tween and 0.1% saponin in 0.1 M Tris and stained for antigens overnight at 4 °C using antibodies in the same blocking solution. The following antibodies and reagents were sued for primary staining; rabbit anti-GATA-3 (EPR16651, ab199428, Abcam, 1:50), anti-CD31 AlexaFlour647 (MEC13.3, 102516, BioLegend, 1:100), anti-CD4 PE (GK1.15, 12-0041-82, Thermo Fisher Scientific, 1:100), anti-F4/80 APC (ab204467, 1:200), anti-MHC Class II eFluor450 (AF6-120.1, 1:100), anti-CXCR6 PE (S051D1, 1:100), Hoescht (1:10,000), rabbit anti-GFP AlexaFlour 488 (polyclonal, A2131, Thermo Fisher Scientific, 1:100). Primary staining was washed out using PBS for 10 min, three washes at RT. Secondary staining (for unconjugated GATA-3) was carried out using donkey anti-rabbit IgG AlexaFlour 488 (A32790, Thermo Fisher Scientific, 1:250) for 1 h at RT and washed out using PBS for 10 min, three washes at RT. Dura was then mounted in Flouromount-G (0100-01, SouthernBiotech) and coverslips applied for imaging. Imaging was carried out using either a Leica TCS SP8 confocal microscope or a Zeiss LSM 880 confocal microscope in super-resolution Airyscan mode with image analysis carried out either in Imaris (Bitplane) or Zeiss ZEN blue, respectively. For quantification of CD4^+^ RORγt^+^ T cells in the dura, manual cell counting was performed using Zeiss ZEN blue for the indicated regions of the dura.

### Bulk TCR-sequencing sample preparation

Perfused dura and intestinal tissues were placed in RNA Later (AM7020, Thermo Fisher Scientific) until RNA extraction with the RNeasy plus micro kit (Qiagen) according to manufacturer’s instructions. Libraries were prepared using SMARTer Mouse TCR a/b Profiling Kit (Takara). Libraries were pooled at equimolar concentration and sequenced on MiSeq (Illumina) on 2× 300-bp run. Resulting libraries were demultiplexed using Casava (Illumina).

### Bulk TCR-sequencing analysis

Fastq files were processed using mixcr (v3.0.13) with the ‘analyze amplicon’ workflow with default settings (--species mmu --starting-material rna --5-end v-primers --3-end j-primers --adapters adapters-present --receptor-type tcr). The output files were then converted using vdjtools (v1.2.1). For each sample, clonotypes were grouped by according to whether sequences displayed identical V and J gene usage and CDR3 amino acid sequences. The clonotypes were then tabulated per treatment per animal per tissue source per locus (TRB or TRA). To tabulate clonotype overlap and sharing, samples were randomly down sampled to the same sequencing depth (100,000 total counts), and the mean percentage overlap (relative to total meningeal clonotype counts) was tabulated over 100 iterations.

### Bulk RNA-seq library construction and sequencing

Perfused dura was placed in RNA Later (AM7020, Thermo Fisher Scientific) until RNA extraction with the RNeasy plus micro kit (Qiagen) according to the manufacturer’s instructions. Genomic DNA contamination was removed using Optimal DNA depletion columns (Qiagen). The quality and concentration of purified RNA was assessed on Bioanalyzer 2000 (Applied Biosystems). Libraries were prepared using the SMARTer stranded total RNA-seq mammalian pico input kit (Takara) according to the manufacturer’s instructions. In total, 5 ng of total RNA was used for the production of libraries. Resulting libraries were reassessed on Bioanalyzer 2000. Libraries were pooled at an equimolar concentration. Sequencing was performed using Hiseq 4000 (Illumina) on a 2× 150-bp sequencing run. Pooled libraries were demultiplexed using Casava (Illumina).

### Bulk RNA-seq processing and analysis

Fastq files from sequencing libraries were trimmed of the first three nucleotides of the R1 strand. Contaminating adaptor sequences, and poor-quality bases were removed using Trim Galore (Babraham Bioinformatics). Libraries were only trimmed for quality. Quality of resulting files were assessed by FastQC and aligned to the mm10 genome using HISAT2, followed by gene-level quantification using the Featurecount function from the Rsubread package in R. All subsequent analysis was performed in R. For within-sample comparisons of chemokine expression, FPKM normalization was applied.

### Single-cell library construction and sequencing

Gene expression libraries were prepared from FACS-sorted populations of single cells as specified in the figures using the 10x Chromium Controller and 10x Chromium Single Cell V(D)J 5′ kit v2 with TCR amplification (10x Genomics). Samples were loaded on to the chip following manufacturer’s recommendations. The resulting library was sequenced on the Illumina NovaSeq 6000 platform (paired-end sequencing, 2× 150 bp). BCL files were demultiplexed using Casava (Illumina). The resulting fastq files were assessed using FastQC (v0.11), FastqScreen (v0.9) and FastqStrand (v0.0.5) before alignment (mm10) and downstream processing with the CellRanger (v7.0) pipeline.

### Processing of scRNA-seq

Single-cell gene expression data from CellRanger count outputs (filtered features, barcodes and count matrices) were analyzed using the Scanpy^61^ (v1.9.1) workflow. Doublet detection was performed using Scrublet^62^ (v0.2.1), with cells from iterative subclustering flagged with outlier Scrublet scores labeled as potential doublets. Cells with counts mapped to >8,000 or <200 genes were filtered. The percentage mitochondrial content cut-off was set at <10.0%. Genes detected in fewer than three cells were filtered. Total gene counts for each cell were normalized to a target sum of 104 and log1p transformed. There resulted in a working dataset of 11,655 cells. Next, highly variable features were selected on the basis of a minimum and maximum mean expression of ≥0.0125 and ≤3 respectively, with a minimum dispersion of 0.5. Total feature counts, mitochondrial percentage and cell cycle (G2M, S phase) scores, were regressed, before scaling. The number of principal components used for neighborhood graph construction was set to 30 initially and subsequently 20 for subgroup analysis. Clustering was performed using the Leiden algorithm with resolution set at 1.0. Uniform manifold approximation and projection (UMAP; v0.5.1) was used for dimensional reduction and visualization, with a minimum distance of 0.3, and all other parameters according to the default settings in Scanpy.

### Analysis of scRNA-seq

Cell types of interest were subset and reclustered iteratively as described above. Resulting clusters were annotated using canonical marker genes and top differentially expressed genes. Differential gene testing was performed using the Wilcoxon rank sum test implemented in Scanpy’s tl.rank_genes_groups. Gene set scoring was performed using Scanpy’s tl.score_genes tool. The gene set representing a tissue-resident lymphocyte signature was obtained from a published RNA-seq dataset^33^. Gene lists for chemokines or chemokine receptors were obtained from the gene ontology (molecular function) database. Gaussian kernel density estimation was performed using Scanpy’s tl.embedding_density function. Unless otherwise indicated, log-transformed expression values were used for plotting. For assessment of *Cxcl16* expression in dural tissues, CNSbordercellatlas.org, an integrated atlas of published scRNA-seq data of the meninges, was downloaded and analyzed^31^. For assessment of chemokine receptor expression on CD4^+^ T cells in the gut, scRNA-seq of sorted colonic CD4^+^ T cells was obtained from GSE160055 and processed and annotated according to the authors’ descriptions^63^. All publicly available data were processed and analyzed in Scanpy, as described above (v1.9.1).

### Processing and analysis of scTCR sequencing

Sequenced single-cell V(D)J (TCR) libraries were first processed with CellRanger vdj, and resulting all_contig.fasta and all_contig_annotations.csv files were re-annotated and processed using the dandelion (v0.2.5) pipeline^64^. TCR contigs were filtered according to cell droplets that passed quality control in the scRNA-seq GEX data, and nonproductive TCR contigs were removed. TCR sequences were assigned to single-cell GEX profiles on the basis of their droplet barcode. Clonal V(D)J clustering was performed on the basis of heavy- and light-chain V–J sequences and junctional sequences. Resulting data were visualized in Scanpy (v1.9.1).

### Figures, data presentation, statistics and data reproducibility

No statistical method was used to predetermine sample size. Samples were excluded if there were technical issues with tissue processing, or if mice had to be culled before the end point. Blinding in mouse experiments was not possible owing to the need to maintain infected mice in separate marked cages from control mice.

Graphs and associated statistical analysis (other than transcriptomic data) were made in GraphPad Prism (v 9.3 and 10.4.2). Where unpaired *t*-tests have been used, data were first checked for normal distribution using the Shapiro–Wilk test; otherwise, Mann–Whitney *U* tests were used. Cartoons and diagrams were made using BioRender. Flow cytometry data were analyzed in FlowJo (v 10.8.1 and v 10.10.0). Where representative flow cytometry plots are given, all effort has been made to ensure they are representative of the mean of the cumulative data where possible.

### Reporting summary

Further information on research design is available in the Nature Portfolio Reporting Summary linked to this article.

## Online content

Any methods, additional references, Nature Portfolio reporting summaries, source data, extended data, supplementary information, acknowledgements, peer review information; details of author contributions and competing interests; and statements of data and code availability are available at https://doi.org/10.1038/s41593-026-02428-4.

## Supplementary information

Reporting summary

## Source data

Source Data Fig. 1 Statistical source data.

Source Data Fig. 2 Statistical source data.

Source Data Fig. 3 Statistical source data.

Source Data Fig. 4 Statistical source data.

Source Data Fig. 5 Statistical source data.

Source Data Fig. 6 Statistical source data.

Source Data Extended Data Fig. 1 Statistical source data.

Source Data Extended Data Fig. 2 Statistical source data.

Source Data Extended Data Fig. 3 Statistical source data.

Source Data Extended Data Fig. 4 Statistical source data.

Source Data Extended Data Fig. 5 Statistical source data.

Source Data Extended Data Fig. 6 Statistical source data.

Source Data Extended Data Fig. 9 Statistical source data.

## Data Availability

Sequencing data have been made available on the GEO public repository under accession numbers GSE233489, GSE233490 and GSE233491. Published scRNA-seq data and accompanying metadata were accessed and downloaded from the GEO repository using the accession GSE160055. Publicly available scRNA-seq data used to identify chemokine receptor expression in sorted murine intestinal CD4^+^ T cells over a range of specified intestinal infections was downloaded from GSE160055. Publicly available scRNA-seq of mouse dural immune cells to visualize CXCL16 expression was downloaded from the CNS border atlas (https://www.cnsbordercellatlas.org) Source data are provided with this paper.

## Code availability

All bulk and single-cell analyses were performed using previously described software packages, which are included in the Methods. No newly generated code or software was used in any part of the data analysis pipeline.

## References

[1] Nabeshima, S., Reese, T. S., Landis, D. M. & Brightman, M. W. Junctions in the meninges and marginal glia. *J. Comp. Neurol.* **164**, 127–169 (1975. 10.1002/cne.901640202 810497

[2] Kilic, T. & Akakin, A. Anatomy of cerebral veins and sinuses. *Front. Neurol. Neurosci.* **23**, 4–15 (2008). 10.1159/000111256 18004050

[3] Schuchardt, F. et al. In vivo analysis of physiological 3D blood flow of cerebral veins. *Eur. Radiol.* **25**, 2371–2380 (2015). 10.1007/s00330-014-3587-x 25638218

[4] Korin, B. et al. High-dimensional, single-cell characterization of the brain’s immune compartment. *Nat. Neurosci.* **20**, 1300–1309 (2017). 10.1038/nn.4610 28758994

[5] Ajami, B. et al. Single-cell mass cytometry reveals distinct populations of brain myeloid cells in mouse neuroinflammation and neurodegeneration models. *Nat. Neurosci.* **21**, 541–551 (2018). 10.1038/s41593-018-0100-x PMC8629134 29507414

[6] Rustenhoven, J. et al. Functional characterization of the dural sinuses as a neuroimmune interface. *Cell* **184**, 1000–1016 (2021). 10.1016/j.cell.2020.12.040 PMC8487654 33508229

[7] Fitzpatrick, Z. et al. Venous-plexus-associated lymphoid hubs support meningeal humoral immunity. *Nature* **628**, 612–619 (2024). 10.1038/s41586-024-07202-9 PMC11482273 38509366

[8] Fitzpatrick, Z. et al. Gut-educated IgA plasma cells defend the meningeal venous sinuses. *Nature* **587**, 472–476 (2020). 10.1038/s41586-020-2886-4 PMC7748383 33149302

[9] Sato, T., Konishi, H., Tamada, H., Nishiwaki, K. & Kiyama, H. Morphology, localization, and postnatal development of dural macrophages. *Cell Tissue Res.* **384**, 49–58 (2021). 10.1007/s00441-020-03346-y 33433687

[10] Filiano, A. J. et al. Unexpected role of interferon-gamma in regulating neuronal connectivity and social behaviour. *Nature* **535**, 425–429 (2016). 10.1038/nature18626 PMC4961620 27409813

[11] Derecki, N. C. et al. Regulation of learning and memory by meningeal immunity: a key role for IL-4. *J. Exp. Med.* **207**, 1067–1080 (2010). 10.1084/jem.20091419 PMC2867291 20439540

[12] Ribeiro, M. et al. Meningeal γδ T cell-derived IL-17 controls synaptic plasticity and short-term memory. *Sci. Immunol.* **4**, eaay5199 (2019). 10.1126/sciimmunol.aay5199 PMC6894940 31604844

[13] Alves de Lima, K. et al. Meningeal γδ T cells regulate anxiety-like behavior via IL-17a signaling in neurons. *Nat. Immunol.* **21**, 1421–1429 (2020). 10.1038/s41590-020-0776-4 PMC8496952 32929273

[14] Peirce, J. M. & Alvina, K. The role of inflammation and the gut microbiome in depression and anxiety. *J. Neurosci. Res.* **97**, 1223–1241 (2019). 10.1002/jnr.24476 31144383

[15] Hsiao, E. Y. et al. Microbiota modulate behavioral and physiological abnormalities associated with neurodevelopmental disorders. *Cell* **155**, 1451–1463 (2013). 10.1016/j.cell.2013.11.024 PMC3897394 24315484

[16] Buffington, S. A. et al. Microbial reconstitution reverses maternal diet-induced social and synaptic deficits in offspring. *Cell* **165**, 1762–1775 (2016). 10.1016/j.cell.2016.06.001 PMC5102250 27315483

[17] Leonardi, I. et al. Mucosal fungi promote gut barrier function and social behavior via type 17 immunity. *Cell* **185**, 831–846 (2022). 10.1016/j.cell.2022.01.017 PMC8897247 35176228

[18] Zhu, J. & Paul, W. E. Peripheral CD4^+^ T-cell differentiation regulated by networks of cytokines and transcription factors. *Immunol. Rev.* **238**, 247–262 (2010). 10.1111/j.1600-065X.2010.00951.x PMC2975272 20969597

[19] Arpaia, N. et al. Metabolites produced by commensal bacteria promote peripheral regulatory T-cell generation. *Nature* **504**, 451–455 (2013). 10.1038/nature12726 PMC3869884 24226773

[20] Furusawa, Y. et al. Commensal microbe-derived butyrate induces the differentiation of colonic regulatory T cells. *Nature* **504**, 446–450 (2013). 10.1038/nature12721 24226770

[21] Constant, S., Pfeiffer, C., Woodard, A., Pasqualini, T. & Bottomly, K. Extent of T cell receptor ligation can determine the functional differentiation of naive CD4^+^ T cells. *J. Exp. Med.* **182**, 1591–1596 (1995). 10.1084/jem.182.5.1591 PMC2192213 7595230

[22] Murphy, K. M. & Reiner, S. L. The lineage decisions of helper T cells. *Nat. Rev. Immunol.* **2**, 933–944 (2002). 10.1038/nri954 12461566

[23] Castro-Dopico, T. et al. Anti-commensal IgG drives intestinal inflammation and type 17 immunity in ulcerative colitis. *Immunity* **50**, 1099–1114 (2019). 10.1016/j.immuni.2019.02.006 PMC6477154 30876876

[24] Collins, J. W. et al. *Citrobacter rodentium*: infection, inflammation and the microbiota. *Nat. Rev. Microbiol.* **12**, 612–623 (2014). 10.1038/nrmicro3315 25088150

[25] Berger, C. N. et al. The *Citrobacter rodentium* type III secretion system effector EspO affects mucosal damage repair and antimicrobial responses. *PLoS Pathog.* **14**, e1007406 (2018). 10.1371/journal.ppat.1007406 PMC6221368 30365535

[26] Pearce, E. J. et al. Th2 response polarization during infection with the helminth parasite *Schistosoma mansoni*. *Immunol. Rev.* **201**, 117–126 (2004). 10.1111/j.0105-2896.2004.00187.x 15361236

[27] Pearce, E. J. & MacDonald, A. S. The immunobiology of schistosomiasis. *Nat. Rev. Immunol.* **2**, 499–511 (2002). 10.1038/nri843 12094224

[28] Else, K. J., Hultner, L. & Grencis, R. K. Modulation of cytokine production and response phenotypes in murine trichuriasis. *Parasite Immunol.* **14**, 441–449 (1992). 10.1111/j.1365-3024.1992.tb00018.x 1437236

[29] Clay, S. L., Bravo-Blas, A., Wall, D. M., MacLeod, M. K. L. & Milling, S. W. F. Regulatory T cells control the dynamic and site-specific polarization of total CD4 T cells following *Salmonella* infection. *Mucosal Immunol.* **13**, 946–957 (2020). 10.1038/s41385-020-0299-1 PMC7567643 32457450

[30] Kiner, E. et al. Gut CD4^+^ T cell phenotypes are a continuum molded by microbes, not by T_H_ archetypes. *Nat. Immunol.* **22**, 216–228 (2021). 10.1038/s41590-020-00836-7 PMC7839314 33462454

[31] Posner, D. A., Lee, C. Y., Portet, A. & Clatworthy, M. R. Humoral immunity at the brain borders in homeostasis. *Curr. Opin. Immunol.* **76**, 102188 (2022). 10.1016/j.coi.2022.102188 35569417

[32] Di Marco Barros, R., Fitzpatrick, Z. & Clatworthy, M. R. The gut–meningeal immune axis: priming brain defense against the most likely invaders. *J. Exp. Med.* **219**, e20211520 (2022). 10.1084/jem.20211520 PMC8932540 35195681

[33] Mackay, L. K. et al. Hobit and Blimp1 instruct a universal transcriptional program of tissue residency in lymphocytes. *Science* **352**, 459–463 (2016). 10.1126/science.aad2035 27102484

[34] Pappalardo, J. L. et al. Transcriptomic and clonal characterization of T cells in the human central nervous system. *Sci. Immunol.* **5**, eabb8786 (2020). 10.1126/sciimmunol.abb8786 PMC8567322 32948672

[35] Steiner, O. et al. Differential roles for endothelial ICAM-1, ICAM-2, and VCAM-1 in shear-resistant T cell arrest, polarization, and directed crawling on blood-brain barrier endothelium. *J. Immunol.* **185**, 4846–4855 (2010). 10.4049/jimmunol.0903732 20861356

[36] Cross, A. & Levine, M. M. Patterns of bacteraemia aetiology. *Lancet Infect. Dis.* **17**, 1005–1006 (2017). 10.1016/S1473-3099(17)30491-7 28818542

[37] Takeshita, N. et al. Unique characteristics of community-onset healthcare- associated bloodstream infections: a multi-centre prospective surveillance study of bloodstream infections in Japan. *J. Hosp. Infect.* **96**, 29–34 (2017). 10.1016/j.jhin.2017.02.022 28377180

[38] Unhanand, M., Mustafa, M. M., McCracken, G. H. Jr. & Nelson, J. D. Gram-negative enteric bacillary meningitis: a twenty-one-year experience. *J. Pediatr.* **122**, 15–21 (1993). 10.1016/s0022-3476(05)83480-8 8419603

[39] van de Beek, D., Brouwer, M. C., Koedel, U. & Wall, E. C. Community-acquired bacterial meningitis. *Lancet* **398**, 1171–1183 (2021). 10.1016/S0140-6736(21)00883-7 34303412

[40] McGill, F. et al. Incidence, aetiology, and sequelae of viral meningitis in UK adults: a multicentre prospective observational cohort study. *Lancet Infect. Dis.* **18**, 992–1003 (2018). 10.1016/S1473-3099(18)30245-7 PMC6105576 30153934

[41] Galvan-Pena, S., Zhu, Y., Hanna, B. S., Mathis, D. & Benoist, C. A dynamic atlas of immunocyte migration from the gut. *Sci. Immunol.* **9**, eadi0672 (2024). 10.1126/sciimmunol.adi0672 PMC10964343 38181094

[42] Sanmarco, L. M. et al. Gut-licensed IFNγ^+^ NK cells drive LAMP1^+^TRAIL^+^ anti-inflammatory astrocytes. *Nature* **590**, 473–479 (2021). 10.1038/s41586-020-03116-4 PMC8039910 33408417

[43] Benakis, C. et al. Commensal microbiota affects ischemic stroke outcome by regulating intestinal γδ T cells. *Nat. Med.* **22**, 516–523 (2016). 10.1038/nm.4068 PMC4860105 27019327

[44] Schnell, A. et al. Stem-like intestinal Th17 cells give rise to pathogenic effector T cells during autoimmunity. *Cell* **184**, 6281–6298 (2021). 10.1016/j.cell.2021.11.018 PMC8900676 34875227

[45] Yoshida, T. M. et al. The subfornical organ is a nucleus for gut-derived T cells that regulate behaviour. *Nature* **643**, 499–508 (2025). 10.1038/s41586-025-09050-7 PMC12768464 40437096

[46] Barberio, B., Zamani, M., Black, C. J., Savarino, E. V. & Ford, A. C. Prevalence of symptoms of anxiety and depression in patients with inflammatory bowel disease: a systematic review and meta-analysis. *Lancet Gastroenterol. Hepatol.* **6**, 359–370 (2021). 10.1016/S2468-1253(21)00014-5 33721557

[47] Smyth, L. C. D. et al. Identification of direct connections between the dura and the brain. *Nature* **627**, 165–173 (2024). 10.1038/s41586-023-06993-7 PMC11254388 38326613

[48] Deol, A. K. et al. Schistosomiasis—assessing progress toward the 2020 and 2025 global goals. *N. Engl. J. Med.* **381**, 2519–2528 (2019). 10.1056/NEJMoa1812165 PMC6785807 31881138

[49] Loveridge, F. G., Ross, W. F. & Blair, D. M. Schistosomiasis; the effect of the disease on educational attainment. *S. Afr. Med. J.* **22**, 260–263 (1948). 18862893

[50] de Clercq, D., Sacko, M., Behnke, J., Gilbert, F. & Vercruysse, J. The relationship between *Schistosoma haematobium* infection and school performance and attendance in Bamako, Mali. *Ann. Trop. Med. Parasitol.* **92**, 851–858 (1998). 10.1080/00034989858899 10396345

[51] Das Sarma, J. et al. Functional interleukin-17 receptor A is expressed in central nervous system glia and upregulated in experimental autoimmune encephalomyelitis. *J. Neuroinflammation* **6**, 14 (2009). 10.1186/1742-2094-6-14 PMC2689857 19400960

[52] Luo, H. et al. Interleukin-17 regulates neuron–glial communications, synaptic transmission, and neuropathic pain after chemotherapy. *Cell Rep.* **29**, 2384–2397 (2019). 10.1016/j.celrep.2019.10.085 31747607

[53] Herz, J. et al. GABAergic neuronal IL-4R mediates T cell effect on memory. *Neuron* **109**, 3609–3618 e3609 (2021). 10.1016/j.neuron.2021.10.022 PMC9116260 34793707

[54] Hanuscheck, N. et al. Interleukin-4 receptor signaling modulates neuronal network activity. *J. Exp. Med.* **219**, e20211887 (2022). 10.1084/jem.20211887 PMC9123307 35587822

[55] Mooney, J. P. et al. Transient loss of protection afforded by a live attenuated non-typhoidal *Salmonella* vaccine in mice co-infected with malaria. *PLoS Negl. Trop. Dis.* **9**, e0004027 (2015). 10.1371/journal.pntd.0004027 PMC4569369 26366739

[56] Ale, A. et al. Model of Host–pathogen Interaction Dynamics Links in Vivo Optical Imaging and Immune Responses. *Infect. Immun*. **85**, 10.1128/iai.00606-16 (2017). PMC5203651 27821583

[57] Martínez-García, E. et al. SEVA 4.0: an update of the Standard European Vector Architecture database for advanced analysis and programming of bacterial phenotypes. *Nucleic Acids Res.* **51**, D1558–D1567 (2022). 10.1093/nar/gkac1059 PMC9825617 36420904

[58] Herrero, M., Lorenzo, V. D. & Timmis, K. N. Transposon vectors containing non-antibiotic resistance selection markers for cloning and stable chromosomal insertion of foreign genes in Gram-negative bacteria. *J. Bacteriol.* **172**, 6557–6567 (1990). 10.1128/jb.172.11.6557-6567.1990 PMC526845 2172216

[59] Ruano-Gallego, D. et al. Type III secretion system effectors form robust and flexible intracellular virulence networks. *Science* **371**, eabc9531 (2021). 10.1126/science.abc9531 33707240

[60] Igyártó, B. Z. et al. Skin-resident murine dendritic cell subsets promote distinct and opposing antigen-specific T helper cell responses. *Immunity* **35**, 260–272 (2011). 10.1016/j.immuni.2011.06.005 PMC3163010 21782478

[61] Wolf, F. A., Angerer, P. & Theis, F. J. SCANPY: large-scale single-cell gene expression data analysis. *Genome Biol.* **19**, 1–5 (2018). 10.1186/s13059-017-1382-0 PMC5802054 29409532

[62] Wolock, S. L., Lopez, R. & Klein, A. M. Scrublet: computational identification of cell doublets in single-cell transcriptomic data. *Cell Syst.* **8**, 281–291 (2019). 10.1016/j.cels.2018.11.005 PMC6625319 30954476

[63] Kiner, E. et al. Gut CD4^+^ T cell phenotypes are a continuum molded by microbes, not by T_H_ archetypes. *Nat. Immunol.* **22**, 216 (2021). 10.1038/s41590-020-00836-7 PMC7839314 33462454

[64] Suo, C. et al. Dandelion uses the single-cell adaptive immune receptor repertoire to explore lymphocyte developmental origins. *Nat. Biotechnol.* **8**, 1–12 (2023). 10.1038/s41587-023-01734-7 PMC10791579 37055623

