# Supplementary material for: Intestinal infections establish antigen-specific, long-lived memory CD4+ T cells in the brain and meninges
Source: Nat Neurosci. 2026 Sep 8;29(11):2741–52. doi: 10.1038/s41593-026-02428-4 (PMC13724277; doi:10.1038/s41593-026-02428-4)
Supplement: Supplementary file 1 [file 41593_2026_2428_MOESM1_ESM.pdf]

nature portfolio

Corresponding author(s): Menna R. Clatworthy

Last updated by author(s): Jul 6, 2026

Reporting Summary

Nature Portfolio wishes to improve the reproducibility of the work that we publish. This form provides structure for consistency and transparency in reporting. For further information on Nature Portfolio policies, see our Editorial Policies and the Editorial Policy Checklist.

Statistics

For all statistical analyses, confirm that the following items are present in the figure legend, table legend, main text, or Methods section.

n/a	Confirmed	
☐	☒	The exact sample size (*n*) for each experimental group/condition, given as a discrete number and unit of measurement
☐	☒	A statement on whether measurements were taken from distinct samples or whether the same sample was measured repeatedly
☐	☒	The statistical test(s) used AND whether they are one- or two-sided<br>*Only common tests should be described solely by name; describe more complex techniques in the Methods section.*
☒	☐	A description of all covariates tested
☐	☒	A description of any assumptions or corrections, such as tests of normality and adjustment for multiple comparisons
☐	☒	A full description of the statistical parameters including central tendency (e.g. means) or other basic estimates (e.g. regression coefficient) AND variation (e.g. standard deviation) or associated estimates of uncertainty (e.g. confidence intervals)
☐	☒	For null hypothesis testing, the test statistic (e.g. *F*, *t*, *r*) with confidence intervals, effect sizes, degrees of freedom and *P* value noted<br>*Give P values as exact values whenever suitable.*
☒	☐	For Bayesian analysis, information on the choice of priors and Markov chain Monte Carlo settings
☒	☐	For hierarchical and complex designs, identification of the appropriate level for tests and full reporting of outcomes
☒	☐	Estimates of effect sizes (e.g. Cohen's *d*, Pearson's *r*), indicating how they were calculated

Our web collection on statistics for biologists contains articles on many of the points above.

Software and code

Policy information about availability of computer code

Data collection	Publicly available scRNA-seq data used to identify chemokine receptor expression in sorted murine intestinal CD4 T cells over a range of specified intestinal infections was downloaded from GSE160055 - Transcriptomic analysis of mouse colon LP CD4+ T cells cells isolated from mouse under different infection conditions<br>Publicly available scRNA-seq of mouse dural immune cells to visualise CXCL16 expression was downloaded from the CNS border atlas (https://www.cnsbordercellatlas.org)<br>The remaining single cell RNA-sequencing and RNA-seq presented in the manuscript was collected in house
Data analysis	Flow cytometry data was analysed with FlowJo (v 10.8, and v 10.10.0)<br>Graphs (other than transcriptomic data) were made in GraphPad Prism (v 9.3, and 10.4.2)<br>Bulk TCR sequencing was performed using mixcr (v3.0.13) and vdjtools (v1.2.1)<br>Single cell sequencing was performed using FastQC (v0.11), FastqScreen (v0.9), FastqStrand (v0.0.5), CellRanger (v.7.0), Scapny (v1.9.1), Scrublet (v0.2.1) and dandelion (v0.2.5)

For manuscripts utilizing custom algorithms or software that are central to the research but not yet described in published literature, software must be made available to editors and reviewers. We strongly encourage code deposition in a community repository (e.g. GitHub). See the Nature Portfolio guidelines for submitting code & software for further information.

Data

Policy information about availability of data

All manuscripts must include a data availability statement. This statement should provide the following information, where applicable:

- Accession codes, unique identifiers, or web links for publicly available datasets
- A description of any restrictions on data availability
- For clinical datasets or third party data, please ensure that the statement adheres to our policy

Sequencing data have been made available on the GEO public repository under accension numbers GSE233489, 233490, and 233491. The data is linked under GEO super-series GSE233544. Published scRNA-seq data and accompanying metadata were accessed and downloaded from the GEO repository using the ascension GSE160055.

Research involving human participants, their data, or biological material

Policy information about studies with human participants or human data. See also policy information about sex, gender (identity/presentation), and sexual orientation and race, ethnicity and racism.

Reporting on sex and gender	Our study did not involve human participants, their data, or biological material
Reporting on race, ethnicity, or other socially relevant groupings	Our study did not involve human participants, their data, or biological material
Population characteristics	Our study did not involve human participants, their data, or biological material
Recruitment	Our study did not involve human participants, their data, or biological material
Ethics oversight	Our study did not involve human participants, their data, or biological material

Note that full information on the approval of the study protocol must also be provided in the manuscript.

Field-specific reporting

Please select the one below that is the best fit for your research. If you are not sure, read the appropriate sections before making your selection.

☒ Life sciences ☐ Behavioural & social sciences ☐ Ecological, evolutionary & environmental sciences

For a reference copy of the document with all sections, see nature.com/documents/nr-reporting-summary-flat.pdf

Life sciences study design

All studies must disclose on these points even when the disclosure is negative.

Sample size	Samples sizes were not predetermined. Sample sizes were chosen based on preliminary studies and on similar effect sizes seen in the field of murine dural immunology. The n number for each individual experiment is presented in the accompanying figure legends and all n numbers are biological replicates unless otherwise specified. Where data was pooled from seperate experiments, it is noted in the accompanying figure legend.
Data exclusions	Samples were excluded if there were technical issues with tissue processing, or if mice had to be culled prior to the end point.
Replication	The n number for each experiment is indicated in the figure legend as well as whether the data is pooled from seperate experiments or representative of independent experiments.
Randomization	Mice were randomised into experimental or control groups
Blinding	Blinding in mouse experiments was not possible due to the need to maintain infected mice in separate marked cages from control mice.

Reporting for specific materials, systems and methods

We require information from authors about some types of materials, experimental systems and methods used in many studies. Here, indicate whether each material, system or method listed is relevant to your study. If you are not sure if a list item applies to your research, read the appropriate section before selecting a response.

Materials & experimental systems

n/a	Involved in the study
☐	☒ Antibodies
☒	☐ Eukaryotic cell lines
☒	☐ Palaeontology and archaeology
☐	☒ Animals and other organisms
☒	☐ Clinical data
☒	☐ Dual use research of concern
☒	☐ Plants

Methods

n/a	Involved in the study
☒	☐ ChIP-seq
☐	☒ Flow cytometry
☒	☐ MRI-based neuroimaging

Antibodies

Antibodies used

Antibodies used for flow cytometry are as follows (Clone, Cat. No, Manufacturer, Dilution used):
anti-CD45 BUV395 (30 F-11, 564279, BD Biosciences, 1:200), CD45.1 BUV395 (A20, 565212, BD Biosciences, 1:200), anti-CD4 BUV496 (GK1.5, 612952, BD Biosciences, 1:200), anti-PD-1 BUV615 (RMP1-30, 752354, BD Biosciences, 1:200), anti-CD19 BUV661 (1D3, 612971, BD Biosciences, 1:200), anti-CD62L BUV737 (MEL-14, 612833, BD Biosciences, 1:200), anti-CD8α BUV805 (53-6.7, 612898, BD Biosciences, 1:200), anti-TCRβ BV421 (H57-597, 109230, BioLegend, 1:100), anti-TNF-α BV421 (MP6-XT22, 506328, BioLegend, 1:200), anti-anti-MHC-II eFlour450 (AF6-120.1, 48-5320-82 eBioscience, 1:400), anti-GATA-3, eFluor450 (TWAJ, 48-9966-42, eBioscience, 1:200), anti-CD45 PacBlue (30-F11, 103126, BioLegend, 1:200), anti-TCRβ BV510 (H57-597, 563221, BioLegend 1:100), anti-CD90.2 BV510 (53-2.1, 140319, BioLegend, 1:400), anti-CD3ε BV605 (145-2C11, 100351 BioLegend, 1:100), anti-CD62L BV605 (MEL-14, 104437 BioLegend, 1:200), anti-CD11b BV650 (M1/70, 101239, BioLegend, 1:300), anti-CD62L BV786 (MEL-14, BioLegend, 564109), anti-Gr1 BV786 (RB6-8C5, 740850 BD Biosciences, 1:400), anti-Ki-67 AF488 (SolA15, 53-5698-82, eBioscience, 1:200), anti-IFN-γ-FITC (XMG1.2, 505806 BioLegend, 1:200), anti-CD45 FITC (30-F11, 103108 BioLegend, 1:200), anti-CD11c FITC (N418, 35-0114 eBioscience, 1:200), anti-CD11b FITC (M1/70, 101206 BioLegend, 1:300), anti-CD3 SparkBlue574 (17A2, 76530-944, VWR, 1:200), anti-CD44 PerCP (IM7, 103036, BioLegend, 1:100), anti-CD19 PerCP (6D5, 115532 BioLegend, 1:200), anti-B220 PerCP-Cy5.5 (RA3-6B2, 103236 BD Biosciences, 1:200), anti-GATA-3 PE (TWAJ, 12-9966-42, eBioscience, 1:200), anti-IL-17a PE (eBio17B7, 12-7177-81 eBioscience, 1:200), anti-T-bet PE (eBio4B10, 12-5825-82, eBioscience, 1:200), anti-CXCL16 PE (12-81, 566740 BD Biosciences, 1:200), anti-CXCR6 PE (SA051D1, 151103 BioLegend, 1:200), anti-Nur77 PE (12.14, 12-5965-82, eBioscience, 1:200), anti-CD69 PE/Dazzle594 (H1.2F3, 104536, BioLegend, 1:200), anti-CD73 PE/Dazzle594 (TY/11.8, 127234, BioLegend,1:200), anti-RORγt PE-Cy7 (B2D, 25-6981-82, eBioscience, 1:200), anti-TCRγδ PE-Cy7 (GL3, 118124 BioLegend, 1:200), anti-Podoplanin PE-Cy7 (8.1.1, 127411 BioLegend, 1:200), anti-CD62L PE-Cy7 (MEL-14, 104418, BioLegend, 1:200), anti-CD45.2 PE-Cy7 (104, 25-0454-82, eBioscience, 1:200), anti-FR4 AF647 (12A5, 125009, BioLegend, 1:200), anti-RORγt APC (B2D, 17-6981-82 eBioscience, 1:200), anti-GATA-3 APC (TWAJ, 50-9966-42 eBioscience, 1:200), anti-CD64 APC (X54-5/7.1, 139306 BioLegend, 1:200), anti-CD45 APC (QA17A26, 157605 BioLegend, 1:200), anti-IFN-γ APC (XMG1.2, 554413, BD Biosciences, 1:200), anti-GATA-3 eFluor660 (TWAJ, eBioscience, 50-9966-42, 1:200), anti-FoxP3 AF700 (FJK-16s, 56-5773-82, eBioscience, 1:200), anti-CD8 APC-Cy7 (YTS156.7.7, 126620 BioLegend, 1:200), anti-CXCR6 APC-Cy7 (SA051D1, 151123, BioLegend, 1:200), anti-CD45 APC-eFluor780 (30-F11, 47-0451-82, Invitrogen, 1:200).

The following were used for confocal imaging (Clone, Cat. No, Manufacturer, Dilution used):
rabbit anti-GATA-3 (EPR16651, ab199428 Abcam, 1:50), anti-CD31 AlexaFlour647 (MEC13.3, 102516 BioLegend, 1:100), anti-CD4 PE (GK1.15, 12-0041-82 ThermoFisher Scientific, 1:100), anti-F4/80 APC (ab204467, 1:200), anti-MHC Class II eFluor 450 (AF6-120.1, 1:100), anti-CXCR6 PE (S051D1,1:100), Hoescht (1:10000), rabbit anti-GFP AlexaFlour 488 (polyclonal, A2131 ThermoFisher Scientific, 1:100), donkey anti-rabbit IgG AlexaFlour 488 (A32790, ThermoFisher Scientific, 1:250).

Validation

All antibodies used in the study are commercially available with relevant validation data provided on the companies website for the listed Cat. No and clone.

Animals and other research organisms

Policy information about studies involving animals; ARRIVE guidelines recommended for reporting animal research, and Sex and Gender in Research

Laboratory animals

Infections were performed in 6-20 week old female WT C57BL/6J mice unless otherwise specified, with the exception of S. mansoni infections (detailed below), and age-matched WT C57BL/6J mice were used as uninfected controls where appropriate.

S. mansoni infections were performed in female BALB/C mice (for confocal imaging) or female TO outbred (HsdOla:TO, Inotiv, UK) mice.

RORγt-eGFP reporter mice were kindly provided by Andrew McKenzie (MRC-LMB, UK).
For the generation of mixed bone marrow chimera, legs from Cxcr6 KO mice were kindly provided by Georg Gasteiger (Universität Würzburg, Germany)

Wild animals

No wild animals were used in this study.

Reporting on sex

Our study was not designed to test sex as a factor in our analysis. Our experiments mainly used female mice

Field-collected samples

Our study used no field-collected samples

Ethics oversight	Experimental protocols were approved by a member of the Animal Welfare and Ethical Review board (AWERB) at the University of Cambridge and the Wellcome Sanger Institute in accordance with the United Kingdom Animals (Scientific Procedures) Act of 1986

Note that full information on the approval of the study protocol must also be provided in the manuscript.

Plants

Seed stocks	*Report on the source of all seed stocks or other plant material used. If applicable, state the seed stock centre and catalogue number. If plant specimens were collected from the field, describe the collection location, date and sampling procedures.*
Novel plant genotypes	*Describe the methods by which all novel plant genotypes were produced. This includes those generated by transgenic approaches, gene editing, chemical/radiation-based mutagenesis and hybridization. For transgenic lines, describe the transformation method, the number of independent lines analyzed and the generation upon which experiments were performed. For gene-edited lines, describe the editor used, the endogenous sequence targeted for editing, the targeting guide RNA sequence (if applicable) and how the editor was applied.*
Authentication	*Describe any authentication procedures for each seed stock used or novel genotype generated. Describe any experiments used to assess the effect of a mutation and, where applicable, how potential secondary effects (e.g. second site T-DNA insertions, mosiacism, off-target gene editing) were examined.*

Flow Cytometry

Plots

Confirm that:

☒ The axis labels state the marker and fluorochrome used (e.g. CD4-FITC).

☒ The axis scales are clearly visible. Include numbers along axes only for bottom left plot of group (a 'group' is an analysis of identical markers).

☒ All plots are contour plots with outliers or pseudocolor plots.

☒ A numerical value for number of cells or percentage (with statistics) is provided.

Methodology

Sample preparation	Mice were perfused post-mortem with ice-cold PBS. For flow cytometry experiments, mice were injected with anti-CD45 flourescent antibody label 5 minutes prior to death, with the exception of experiments involving S. mansoni infections, CXCL16 production or ex vivo peptide stimulation assays where mice were perfused with ice-cold PBS.<br><br>Spleens were mechanically disaggregated through a 70 µm cell filter using the rubber end of a 1mL syringe insert. Cells were washed with 3% PBS/FCS and resuspended in 2mL RBC lysis buffer for 1 min. Lysis was quenched with 3% FCS PBS. 1/10 of spleen samples were used for flow cytometry staining.<br>Skull cap dura was dissected in ice-cold PBS under a dissecting microscope, transferred to 1ml digest medium (1mL RPMI, 2% FCS, 10mM HEPES, 2.5mg/mL Collagenase D [Thermo Fisher Scientific, 11088866001], 125 µg/mL DNAse I [Merck, 10104159001]), and incubated for 30min at 37°C with gentle agitation at 15 min. Tissue was vortexed vigorously, pipetted up and down, and passed through a 70 µm FACS tube filter.<br>For colon, Extraneous fat was removed using curved scissors and faecal content extruded using forceps. The colon was opened longitudinally, washed in ice-cold PBS/HEPES, and cut into 0.5cm pieces for transfer into epithelial stripping buffer (5mL RPMI, 2% FCS, 10 mM HEPES, 5mM EDTA, 1mM DTT). This was incubated at 37◦C shaking for 25 min, vortexed, and washed in PBS/HEPES through a wire-mesh filter before transfer into digest buffer (5mL RPMI, 2% FCS, 10 mM HEPES, 1 mg/mL collagenase I [Thermo Fisher Scientific, Cat. No 17100017], 100 µg/mL DNAse I [Merck, Cat. No 10104159001]). Digestion was performed with shaking at 37°C for 30-40 min followed by vortex. Homogenates were passed through a 70 µm cell filter, mashed using the rubber end of a 1mL syringe insert, and washed with 3% FCS/PBS. Immune cell-enriched suspensions were obtained via percoll-density centrifugation (20mins, 600 x g). Cells were harvested from the 40:80% interface.<br><br>Staining was carried out in 96 well V-bottom plates (Thermo Scientific Fisher, 612V96). Precision Count Beads (BioLegend, 424902) were added to enumerate total cell number. Live:dead discrimination was achieved using ViaKrome 808 Fixable Viability Dye (Beckmann Coulter, C36628) or Live/Dead Near-IR, and blocking was performed using 3% PBS/FCS containing 0.5% (v/v) heat-inactivated normal mouse/rat serum (Invitrogen, 10410/Thermo Scientific Fisher, 10-710-C) prior to antibody and/or tetramer staining.
Instrument	Flow cytometry analysis was performed on a 6-laser CytoFLEX LX Flow Cytometer (Beckman Coulter), or a 5-laser Cytek Aurora Spectral flow cytometer (Cytek Biosciences)<br>Flow cytometry sorting was performed on a BD Influx
Software	FCS files were analysed using FlowJo (v10.8, or v 10.10)
Cell population abundance	Pre- and post-sorting plots for sorted dural extravascular CD45+ cells are provided in Extended data Figure 7b.
Gating strategy	Gating strategies are provided in Extended data Figure 1 f-e and Extended data Figure 5c.

☒ Tick this box to confirm that a figure exemplifying the gating strategy is provided in the Supplementary Information.
